# Olverembatinib (HQP1351), a well-tolerated and effective tyrosine kinase inhibitor for patients with T315I-mutated chronic myeloid leukemia: results of an open-label, multicenter phase 1/2 trial

**DOI:** 10.1186/s13045-022-01334-z

**Published:** 2022-08-18

**Authors:** Qian Jiang, Zongru Li, Yazhen Qin, Weiming Li, Na Xu, Bingcheng Liu, Yanli Zhang, Li Meng, Huanling Zhu, Xin Du, Suning Chen, Yang Liang, Yu Hu, Xiaoli Liu, Yongping Song, Lichuang Men, Zi Chen, Qian Niu, Hengbang Wang, Ming Lu, Dajun Yang, Yifan Zhai, Xiaojun Huang

**Affiliations:** 1 National Clinical Research Center for Hematologic Disease, Peking University People’s Hospital, Peking University Institute of Hematology, No. 11 South Street of Xizhimen, Xicheng District, Beijing, 100044 China; 2 Union Hospital, Tongji Medical College, Huazhong University of Science and Technology, Wuhan, 430022 Hubei China; 3 Department of Hematology, Nanfang Hospital, Southern Medical University, 1838 Guangzhou N Ave, Baiyun, Guangzhou, 510515 Guangdong Province China; 4 State Key Laboratory of Experimental Hematology, National Clinical Research Center for Blood Diseases, Haihe Laboratory of Cell Ecosystem, Institute of Hematology & Blood Diseases Hospital, Chinese Academy of Medical Sciences & Peking Union Medical College, Tianjin, 300020 China; 5 Department of Hematology, The Affiliated Cancer Hospital of Zhengzhou University and Henan Cancer Hospital, Zhengzhou, 450008 Henan China; 6 Department of Hematology, Tongji Hospital of Tongji Medical College, Huazhong University of Science and Technology, Wuhan, 430022 Hubei China; 7 Department of Hematology, West China Hospital of Sichuan University, No. 37 Guoxue Alley, Wuhou District, Chengdu City, 610000 Sichuan Province China; 8 Division of Hematology, Shenzhen Second People’s Hospital, The First Affiliated Hospital of Shenzhen University, 3002 Sungang W Rd, Futian District, Shenzhen, 518000 Guangdong Province China; 9 National Clinical Research Center for Hematologic Diseases, The First Affiliated Hospital of Soochow University, Jiangsu Institute of Hematology, Institute of Blood and Marrow Transplantation, Collaborative Innovation Center of Hematology, Soochow University, Suzhou, China; 10 State Key Laboratory of Oncology in South China, Department of Hematologic Oncology, Collaborative Innovation Center for Cancer Medicine, Sun Yat-Sen University Cancer Center, 651 Dongfeng Road East, Guangzhou, 510060 Guangdong Province China; 11 Guangzhou Healthquest Pharma Co. Ltd., Room F314, GIBI, No. 3 Lanyue Road, Guangzhou, 510663 China; 12 Ascentage Pharma Group Inc., 800 King Farm Blvd Suite 300, Rockville, MD 20850 USA; 13 Ascentage Pharma (Suzhou) Co., Ltd, 218 Xinghu St, Bldg B7, 7th Floor, Suzhou Industrial Park, Suzhou, 215000 Jiangsu China; 14 State Key Laboratory of Oncology in South China, Collaborative Innovation Center for Cancer Medicine, 651 Dongfeng Road East, Guangzhou, 510060 Guangdong Province China; 15 Beijing Key Laboratory of Hematopoietic Stem Cell Transplantation, No. 11 South Street of Xizhimen, Xicheng District, Beijing, 100044 China; 16 Peking-Tsinghua Center for Life Sciences, No. 11 South Street of Xizhimen, Xicheng District, Beijing, 100044 China; 17 Academy for Advanced Interdisciplinary Studies, Peking University, No. 11 South Street of Xizhimen, Xicheng District, Beijing, 100044 China

**Keywords:** Chronic myeloid leukemia, Chronic phase, Accelerated phase, T315I mutation, Tyrosine kinase inhibitor

## Abstract

**Background:**

*BCR-ABL1*^*T315I*^ mutations confer resistance to tyrosine kinase inhibitors (TKIs) in chronic myeloid leukemia (CML). Olverembatinib is a new potent *BCR-ABL1* TKI with preclinical activity against T315I-mutated CML. In phase 1/2 studies, we explored the safety and efficacy of olverembatinib in Chinese adults with TKI-resistant CML in the chronic phase (CML-CP) and accelerated phase (CML-AP).

**Methods:**

In the phase 1 study, olverembatinib was orally administered once every other day in 28-day cycles at 11 dose cohorts ranging from 1 to 60 mg, and we evaluated the maximum tolerated dose, recommended phase 2 dose (RP2D), safety, efficacy, and pharmacokinetics of olverembatinib. In the phase 2 studies, olverembatinib was administered at the RP2D of 40 mg orally on alternate days for 28-day cycles. The primary outcome measure is major cytogenetic response (MCyR) and major hematologic response by the end of Cycle 12 in CML-CP and CML-AP, respectively. Fine and Gray's hazard models were used to identify covariates associated with responses.

**Results:**

A total of 165 patients (> 80.0% of whom had received ≥ 2 TKIs) were enrolled in this study. Among 127 patients with CML-CP, the 3-year cumulative incidences of achieving MCyR, complete cytogenetic response (CCyR), major molecular response (MMR), MR^4.0^, and MR^4.5^ were 79.0, 69.0, 56.0, 44.0 and 39.0%, respectively. The highest response rates were observed in patients with a single T315I mutation. Among 38 patients with CML-AP, the 3-year cumulative incidences of achieving MCyR, CCyR, MMR, MR^4.0^, and MR^4.5^ were 47.4%, 47.4%, 44.7%, 39.3%, and 32.1%, respectively. In multivariate analyses, baseline *BCR-ABL1* mutation status was significantly associated with cytogenetic and molecular responses. Common treatment-related adverse events included skin hyperpigmentation, hypertriglyceridemia, proteinuria, and severe thrombocytopenia.

**Conclusions:**

Olverembatinib was well tolerated, with significant antileukemic activity in adults with TKI-resistant CML-CP and CML-AP, especially those with the T315I mutation.

*Trial registration*: The phase 1 trial is registered at CTR20220566, and the two single-arm, open-label phase 2 studies are registered at ClinicalTrials.gov: NCT03883087 (CML-CP) and NCT03883100 (CML-AP).

**Supplementary Information:**

The online version contains supplementary material available at 10.1186/s13045-022-01334-z.

## Background

Introduction of tyrosine kinase inhibitors (TKIs) has revolutionized the treatment of chronic myeloid leukemia (CML) [1–3], but resistance to TKIs remains a challenge. The *BCR-ABL1*^*T315I*^ “gatekeeper” (the most common kinase domain) mutation confers a high degree of resistance to imatinib and all second-generation (2G) TKIs [4]. Before the advent of third-generation (3G) TKIs such as ponatinib, this mutation was associated with rapid progression and limited survival [5].

Ponatinib and the allosteric BCR-ABL1 (Specifically Targeting the ABL Myristoyl Pocket [STAMP]) inhibitor asciminib are recommended for patients with the *BCR-ABL1*^*T315I*^ mutation [6]. However, a subset of patients cannot achieve desired treatment outcomes[Cortes, 2018 #4707;Cortes, 2021 #4704;Cortes, 2013 #4706]. Certain drug-related adverse events (AEs) also present a formidable challenge despite dose optimization, including arterial occlusive events with ponatinib [7, 8]. In addition, ponatinib and asciminib are not available worldwide, particularly in emerging regions. It is thus an urgent unmet need to develop an alternative TKI that is efficacious and well tolerated.

Olverembatinib (HQP1351) is a novel, small-molecule, orally administered 3G BCR-ABL1 inhibitor designed with scaffold-hopping strategies that culminate in extensive donor–acceptor hydrogen bond networks [9, 10] (Additional file 1: Fig. 1). This innovative TKI exhibited potent in vitro inhibitory activity against both wild-type and mutant BCR-ABL1 (IC_50_ 0.5 nM) and enhanced cell cycle arrest and apoptosis in CML cells [11]. Olverembatinib also induced regression of subcutaneous CML cells expressing wild-type and mutant *BCR-ABL1* (including T315I) in studies of xenograft and allograft animal models [9, 10]. The aim of the current study was to assess the efficacy, safety, and pharmacology of olverembatinib in patients with TKI-resistant chronic-phase or accelerated phase CML (CML-CP or CML-AP).

## Methods

### Study design

This was a phase 1/2, open-label, two-part study. Part 1 was a phase 1 “3 + 3” dose escalation and expansion study using a standard design, with 11 successive cohorts of TKI-resistant CML patients receiving oral olverembatinib at increasing alternate-day (QOD) doses of 1–60 mg. During dose escalation, the starting dose of olverembatinib was 1 mg administered orally QOD (Additional file 1: Fig. 2). Each cohort was assessed for safety over a treatment cycle of 28 days before enrolling the next cohort, in order to identify the maximum tolerated dose (MTD), or the dose at which the dose-limiting toxicity (DLT) occurred in no more than 33.0% of patients during the first cycle. DLT was defined as any of the following during the first 28 days of olverembatinib therapy (Cycle 1): (1) grade 3 or higher (G3^+^) nonhematologic AEs exceeding 3 days despite adequate supportive care; (2) missed doses due to toxicity (for > 25% of the first cycle); (3) febrile neutropenia unrelated to leukemia; or (4) G4 cytopenia (for > 28 days but unrelated to underlying disease, with < 5% cellularity in bone marrow). In the event of a lack of efficacy, the study permitted intrapatient dose escalation unless the olverembatinib regimen exceeded MTD. After that, dose expansion cohorts at targeted doses were initiated. The primary endpoint within the phase 1 study was the recommended phase 2 dose (RP2D), and the Chinese Clinical Trial Registry (www.chictr.org.cn) identifier was ChiCTR1900027568.

Part two was a phase 2 study evaluating the efficacy and safety of olverembatinib at RP2D in patients with *T315I*-mutated CML-CP and CML-AP. The primary endpoints of the phase 2 study were major cytogenetic response (MCyR) in patients with CML-CP and major hematologic response (MaHR) in those with CML-AP. In this phase 2 study, two pivotal open-label single-arm multicenter studies were performed (clinicaltrials.gov identifiers NCT03883087 for the CML-CP study and NCT03883100 for the CML-AP study) and included adults with *BCR-ABL1*^*T315I*^-driven TKI resistance.

There were two other major differences between parts 1 and 2 of this study: (A) part 1 enrolled patients with a range of *BCR-ABL1* genotypes, whereas part 2 enrolled only patients with the T315I “gatekeeper” mutation; and (B) part 1 enrolled patients with third-line (3L) or higher treatment, whereas part 2 also included patients with second-line (2L) TKIs because the T315I mutation confers resistance to all first- and second-generation TKIs.

### Patient population

Eligible participants were adults with Philadelphia chromosome (Ph)- or *BCR-ABL1-*positive CML-CP or CML-AP resistant to standard care in China, with disease phases diagnosed by 2013 European LeukemiaNet (ELN) criteria [12]. (Inclusion and exclusion criteria are summarized in Additional file 1: Table 1) In brief, parts 1 and 2 both included adults who had: (1) TKI-resistant or refractory Philadelphia chromosome-positive and/or BCR-ABL1 fusion gene positive CML,

(2) Eastern Cooperative Oncology Group performance status ≤ 2, and (3) adequate organ function but not a range of conditions that can complicate TKI treatments, including cardiovascular and pulmonary vascular disorders. Part 1 included patients with a range of *BCR-ABL1* genotypes, whereas Part 2 included only those with the T315I (“gatekeeper") mutation.

### Safety assessments

Adverse events were assessed continuously and graded according to NCI CTCAE v5.0.18. Complete blood count with differential, serum chemistries, and physical examinations were performed before the study; weekly during Cycles 1 and 2; on alternate weeks in Cycles 3 and 4; and monthly thereafter.

### Efficacy assessments

Hematologic response to olverembatinib was evaluated before study entry and on Day 1 of each treatment cycle. Morphologic assessments and cytogenetic analyses using bone marrow were performed in local laboratories. Quantitative reverse-transcription polymerase chain reaction (RT-qPCR) assays of *BCR-ABL1* transcripts were performed on blood in two central laboratories before study entry and every 3 months thereafter (but every 2 months in phase 2 subjects with CML-AP) [13–16].

Sanger sequencing to identify *BCR-ABL1* kinase domain mutations was performed in all patients before enrollment, while next-generation sequencing (NGS) via the Pacific Biosciences (PacBio®; Menlo Park, CA) RSII system detailed in previous studies [17] was used to identify *BCR-ABL1* compound mutations (defined as ≥ 2 mutations in the same BCR-ABL1 molecule [18]) at Peking University People’s Hospital. Mutational analyses of the PacBio data were performed using custom python scripts. To control for false positives, the detection threshold for a single mutation site was set to 3.0% based on pre-experimental data. If the frequency of compound mutations exceeded 3.0%, they were considered to be true compound mutations. *BCR-ABL1* splice isoforms were identified from full-length circular consensus sequence reads spanning the entire transcript.

Hematologic, cytogenetic, and molecular response criteria were based on 2013 ELN recommendations [12], including complete hematologic response (CHR); major cytogenetic response (MCyR) or complete cytogenetic response (CCyR); as well as major molecular response (MMR), MR^4.0^, and MR^4.5^. Major hematologic response (MaHR) was defined as CHR or no evidence of leukemia and assessed in patients with CML-AP [12]. Progression included advances from CML-CP to CML-AP or blast phase CML (CML-BP) on olverembatinib.

### Pharmacokinetics and pharmacodynamics

The phase 1 study included pharmacokinetic analyses, for which blood samples were collected on Cycle 1 Day 1–2 (C1D1-C1D2) and C1D27-C1D28. Blood samples were collected on C1D1 before dosing (baseline) as well as at 8 and 24 h after dosing on C1D1, C1D15, and C1D27. For pharmacodynamic evaluations, phosphorylated CRK-like (pCRKL) adaptor protein in peripheral blood mononuclear cells was assayed as a biomarker of BCR-ABL1 inhibition.

### Statistical analyses

Patients receiving ≥ 1 dose of olverembatinib were included in safety analyses. Patients who did not reach a given endpoint at baseline were evaluated for responses on treatment. Response rates and two-sided exact (Clopper-Pearson) 95% confidential interval [CI] were calculated. Fine and Gray's model were used to identify co-variates associated with cytogenetic and molecular responses. Treatment discontinuation before response was considered a competing risk.

Progression-free survival (PFS) was calculated from the start date of an effective dose to progression, death, or censorship at last contact. Overall survival (OS) was calculated from the start date of an effective dose to all-cause death or censorship at last follow-up. The Kaplan–Meier method was used to estimate durations of sustained response, PFS, and OS. Post hoc analyses were performed without multiple comparison adjustments, and nominal *P* values were reported. All statistical analyses were performed using SAS software version 9.4 (SAS Institute, Cary, NC).

## Results

### Patient characteristics

Between October 26, 2016, and October 8, 2019, 165 patients were enrolled: 127 with CML-CP and 38 with CML-AP**.** A total of 110 (66.7%) patients were men. The median age was 42 (range, 20–74) years (Table 1) and median interval from CML diagnosis to first olverembatinib dose.

**Table 1:** Patient characteristics

	Total	Chronic phase CML	Accelerated phase CML
Patient number	165	127	38
Age (y), median (range)	42 (20–74)	43 (20–70)	38.5 (21–74)
Male, n (%)	110 (66.7)	79 (62.2)	31 (81.6)
*ECOG performance status, n (%)*			
0	99 (60.0)	82 (64.6)	17 (44.7)
1	64 (38.8)	43 (33.9)	21 (55.3)
Not done	2 (1.2)	2 (1.6)	0
Time from diagnosis to olverembatinib treatment (y), median (range)	5.7 (0.3–23.2)	5.3 (0.6–23.2)	6.9 (0.3–14.7)
*Prior TKIs, n (%)*			
Imatinib	22 (13.3)	16 (12.6)	6 (15.8)
Imatinib/dasatinib	60 (36.4)	47 (37.0)	13 (34.2)
Imatinib/nilotinib	26 (15.8)	22 (17.3)	4 (10.5)
Imatinib/dasatinib/nilotinib	45 (27.3)	35 (27.6)	10 (26.3)
Nilotinib	6 (3.6)	4 (3.1)	2 (5.3)
Dasatinib	2 (1.2)	1 (0.8)	1 (2.6)
Dasatinib/nilotinib	4 (2.4)	2 (1.6)	2 (5.3)
*Number of lines of prior TKI therapy, n (%)*			
1	30 (18.2)	21 (16.5)	9 (23.7)
2	90 (54.5)	71 (55.9)	19 (50.0)
≥ 3	45 (27.3)	35 (27.6)	10 (26.3)
*BCR-ABL1 mutation status by Sanger sequencing, n (%)*			
No mutation	24 (14.5)	23 (18.1)	1 (2.6)
T315I single mutation	102 (61.8)	77 (60.6)	25 (65.8)
T315I + additional mutations	25 (15.2)	16 (12.6)	9 (23.7)
Other mutations	14 (8.5)	11 (8.7)	3 (7.9)
*BCR-ABL1 mutation status by next-generation sequencing, n (%)*			
No mutation	20 (16.9)	19 (20.2)	1 (4.2)
T315I single mutation	53 (44.9)	41 (43.6)	12 (50.0)
T315I + additional mutations	19 (16.1)	15 (16.0)	4 (16.7)
Other mutations	14 (11.9)	12 (12.8)	2 (8.3)
Compound mutations	12 (10.2)	7 (7.4)	5 (20.8)
*ACA, n (%)*			
Yes	28 (17.0)	10 (7.9)	18 (47.4)
No	137 (83.0)	117 (92.1)	20 (52.6)

*ACA* additional chromosomal abnormalities, *CML* chronic myeloid leukemia, *ECOG* Eastern Cooperative Oncology Group, *TKI* tyrosine kinase inhibitor

5.7 (range, 0.3–23.2) years. A total of 30 (18.2%) patients had received 1 prior TKI (Additional file 1: Table 5), 90 (54.5%) had 2 prior TKIs, and 45 (27.3%) at least 3. These second-line patients were predominantly male and in chronic phase (70% each), with a median time from diagnosis to olverembatinib treatment of 1.6 years and of 28 of them had *T315I* mutation identified by Sanger sequencing.

Sanger sequencing identified 102 (61.8%) patients with a single T315I mutation, 25 (15.2%) with T315I and additional mutations (Additional file 1: Table 6), 14 (8.5%) with other mutations, and 24 (14.5%) with no *BCR-ABL1* mutation. Corresponding data in 118 patients, (94 CML-CP and 24 CML-AP) receiving NGS were 53 (44.9%; single T315I mutation), 19 (16.1%; T315I and additional noncompound mutations), 14 (11.9%; other noncompound mutations), 12 (10.2%; compound mutations), and 20 (16.9%; no *BCR-ABL1* mutation). Additional mutations observed in conjunction with T315I included phosphate-binding loop (P-loop) residue E255 (which confers resistance against imatinib, nilotinib, and bosutinib), C-lobe residue F359 (nilotinib), and gatekeeper F317 (dasatinib).

### Phase 1 study

A total of 101 patients with TKI-resistant CML were enrolled in the phase 1 study between October 26, 2016, and December 12, 2018. Among these 101 patients, 86 had CML-CP and 15, CML-AP. Demographic and baseline disease characteristics are summarized in Additional file 1: Table 7. Sanger sequencing identified 46 (45.5%) patients with a single T315I mutation, 17 (16.8%) with T315I and additional mutations, 14 (13.9%) with other mutations, and 24 (23.8%) with no *BCR-ABL1* mutation. Corresponding data in 94 patients (81 CML-CP and 13 CML-AP) receiving NGS were 33 (35.1%; single T315I mutation), 16 (17.0%; T315I and additional noncompound mutations), 13 (13.8%; other noncompound mutations), 12 (12.8%; compound mutations), and 20 (21.3%; no *BCR-ABL1* mutation). Across 11 dose cohorts (1 to 60 mg QOD) in 28-day cycles, no DLT was observed at doses below 60 mg. Two of three patients in the 60 mg cohort experienced DLTs, including 1 G4 thrombocytopenia and 1 myocardial injury, which resulted in dose interruption. Among 33 patients in dose escalation, the MTD was 50 mg. Based on preliminary safety and efficacy results, we expanded the 30, 40, and 50 mg QOD dose cohorts. Finally, the RP2D was established as 40 mg QOD.

### Phase 2 study

After determination of RP2D, two pivotal studies were initiated at 10 sites in China from April 26, 2019, to October 8, 2019, that enrolled 41 patients with T315I-mutated CML-CP and 23 with T315I-mutated CML-AP. Demographic and baseline disease characteristics are summarized in Additional file 1: Table 7. More than 60% of patients were treated in second-line therapy. Sanger sequencing identified 37 (90.2%) patients with a single T315I mutation and 4 (9.8%) with T315I and additional mutations in CML-CP; while patients with CML-AP were 19 (82.6%) with a single T315I mutation and 4 (17.4%) with T315I and additional mutations. Corresponding data in 24 patients (13 CML-CP and 11 CML-AP) receiving NGS were 11 (84.6%) with a single T315I mutation, 1 (7.7%) with T315I and additional noncompound mutations, and 1 (7.7%) with other noncompound mutations in patients with CML-CP, as well as 9 (81.8%; single T315I mutation) and 2 (18.2%; T315I and additional noncompound mutations) in those with CML-AP.

### Patient disposition

By September 30, 2021, the median follow-up was 34.3 (range, 4.8–58.6) months. A total of 114 (69.0%) patients remained on treatment at doses of 20 (*n* = 11), 30 (*n* = 44), 40 (*n* = 48), or 50 mg (*n* = 11) QOD (Table 2). Treatment interruptions due to AEs occurred in 86 (52.1%) patients, including 58 (45.7%) with CML-CP and 28 (73.7%) with CML-AP. Median number and duration of treatment interruptions were 2 (range, 1–11) and 56 (range, 1–421) days.

**Table 2:** Patient disposition

	Total	Chronic phase CML	Accelerated phase CML
Patient number	165	127	38
Treatment duration (mo.), median (range)	30.7 (1.2–58.6)	34.5 (1.2–57.5)	25.5 (1.4–58.6)
Ongoing, *n* (%)	114 (69.1)	96 (75.6)	18 (47.4)
Olverembatinib 20 mg	11 (9.6)	8 (8.3)	3 (16.7)
Olverembatinib 30 mg	44 (38.6)	38 (39.6)	6 (33.3)
Olverembatinib 40 mg	48 (42.1)	41 (42.7)	7 (38.9)
Olverembatinib 50 mg	11 (9.6)	9 (9.4)	2 (11.1)
Discontinuation, n (%)	51 (30.9)	31 (24.4)	20 (52.6)
Disease progression	15 (9.1)	5 (3.9)	10 (26.3)
Adverse events	13 (7.9)	9 (7.1)	4 (10.5)
Treatment failure	12 (7.2)	8 (6.3)	4 (10.5)
Withdrawal by subject	8 (4.8)	8 (6.3)	0
Death	3 (1.8)	1 (0.8)	2 (5.3)

*CML* chronic myeloid leukemia

Doses were reduced because of AEs in 50 (30.3%) patients, including 36 (28.3%) with CML-CP and 14 (36.8%) with CML-AP. The most common AE leading to dose reduction was severe thrombocytopenia (*n* = 26; 15.8%) (Additional file 1: Table 2). Fifty-one patients permanently discontinued treatment because of either CML progression (*n* = 15; 9.1%), AEs (*n* = 13; 7.9%), treatment failure (*n* = 12; 7.0%), consent withdrawal (*n* = 8; 4.8%), or death (*n* = 3; 1.8%) (Table 2).

### Safety

The median treatment duration was 30.7 (range, 1.2–58.6) months. All 165 patients experienced at least 1 treatment-related AE (TRAE), of which 131 (79.4%) were G3/4 (Table 3). The most frequent nonhematologic TRAE was skin hyperpigmentation in 139 (84.2%) patients with pathologically confirmed lentiginous nevus in 2 patients, followed by hypertriglyceridemia (57.6%), proteinuria (50.9%), hyperbilirubinemia (41.8%), hypocalcemia (38.8%), and elevated liver transaminases (35.8%). Median time to onset of these TRAEs was 70 (range, 1–1,315) days.

**Table 3:** Treatment-related adverse events (≥ 10%, all grade)

	Total		Chronic phase CML		Accelerated phase CML	
Patient number	165		127		38	
Event, *n* (%)	Any grades	G 3/4	Any grades	G 3/4	Any grades	G 3/4
Treatment-related adverse events	165 (100.0)	131 (79.4)	127 (100.0)	97 (76.4)	38 (100.0)	34 (89.5)
Nonhematologic	165 (100.0)	81 (49.1)	127 (100.0)	61 (48.0)	38 (100.0)	20 (52.6)
Skin pigmentation	139 (84.2)	0	108 (85.0)	0	31 (81.6)	0
Hypertriglyceridemia	95 (57.6)	12 (7.3)	72 (56.7)	11 (8.7)	23 (60.5)	1 (2.6)
Proteinuria	84 (50.9)	6 (3.6)	65 (51.2)	5 (3.9)	19 (50.0)	1 (2.6)
Hyperbilirubinemia	69 (41.8)	4 (2.4)	56 (44.1)	3 (2.4)	13 (34.2)	1 (2.6)
Hypocalcemia	64 (38.8)	0	45 (35.4)	0	19 (50.0)	0
Increased alanine aminotransferase	59 (35.8)	4 (2.4)	50 (39.4)	3 (2.4)	9 (23.7)	1 (2.6)
Increased aspartate aminotransferase	59 (35.8)	4 (2.4)	49 (38.6)	2 (1.6)	10 (26.3)	2 (5.3)
Increased creatine phosphokinase	48 (29.1)	11 (6.7)	43 (33.9)	10 (7.9)	5 (13.2)	1 (2.6)
Increased γ-glutamyl transferase	47 (28.5)	5 (3.0)	38 (29.9)	5 (3.9)	9 (23.7)	0
Hyperglycemia	43 (26.1)	1 (0.6)	33 (26.0)	1 (0.8)	10 (26.3)	0
Hypokalemia	42 (25.5)	2 (1.2)	32 (25.2)	1 (0.8)	10 (26.3)	1 (2.6)
Hyponatremia	38 (23.0)	1 (0.6)	28 (22.0)	1 (0.8)	10 (26.3)	0
Myalgia	31 (18.8)	0	23 (18.1)	0	8 (21.1)	0
Pyrexia	30 (18.2)	9 (5.5)	21 (16.5)	5 (3.9)	9 (23.7)	4 (10.5)
Sinus tachycardia	30 (18.2)	0	23 (18.1)	0	7 (18.4)	0
Rash	27 (16.4)	1 (0.6)	19 (15.0)	1 (0.8)	8 (21.1)	0
Hypoalbuminemia	26 (15.8)	0	20 (15.7)	0	6 (15.8)	0
Hyperphosphatemia	22 (13.3)	0	11 (8.7)	0	11 (28.9)	0
Hypertension	22 (13.3)	9 (5.5)	19 (15.0)	7 (5.5)	3 (7.9)	2 (5.3)
Increased lipase	22 (13.3)	5 (3.0)	16 (12.6)	5 (3.9)	6 (15.8)	0
Increased alkaline phosphatase	21 (12.7)	1 (0.6)	16 (12.6)	1 (0.8)	5 (13.2)	0
Pain in extremity	21 (12.7)	2 (1.2)	14 (11.0)	1 (0.8)	7 (18.4)	1 (2.6)
Arthralgia	19 (11.5)	1 (0.6)	11 (8.7)	0	8 (21.1)	1 (2.6)
Asthenia	18 (10.9)	0	12 (9.4)	0	6 (15.8)	0
Hematologic	138 (83.6)	93 (56.4)	102 (80.3)	67 (52.8)	36 (94.7)	26 (68.4)
Thrombocytopenia	126 (76.4)	85 (51.5)	93 (73.2)	62 (48.8)	33 (86.8)	23 (60.5)
Anemia	90 (54.5)	38 (23.0)	66 (52.0)	26 (20.5)	24 (63.2)	12 (31.6)
Leukopenia	56 (33.9)	34 (20.6)	37 (29.1)	21 (16.5)	19 (50.0)	13 (34.2)
Neutropenia	26 (15.8)	19 (11.5)	18 (14.2)	11 (8.7)	8 (21.1)	8 (21.1)

Cardiovascular events (CVEs) possibly related to olverembatinib were observed in 53 (32.1%) patients at a median of 11 (range, 0.03–53) months on treatment, including hypertension (13.3%), pericardial effusion (8.5%), ventricular extrasystoles (4.2%), supraventricular extrasystoles or atrial fibrillation (3.0% each), retinal-vein occlusion (1.8%) or palpitations (1.2%); as well as angina pectoris, arrhythmia, atrial tachycardia, cardiomegaly, cerebral ischemia, and/or cerebral infarction in < 1%; of which 11.5% were G3/4 (Additional file 1: Table 3).

**Fig. 1:**
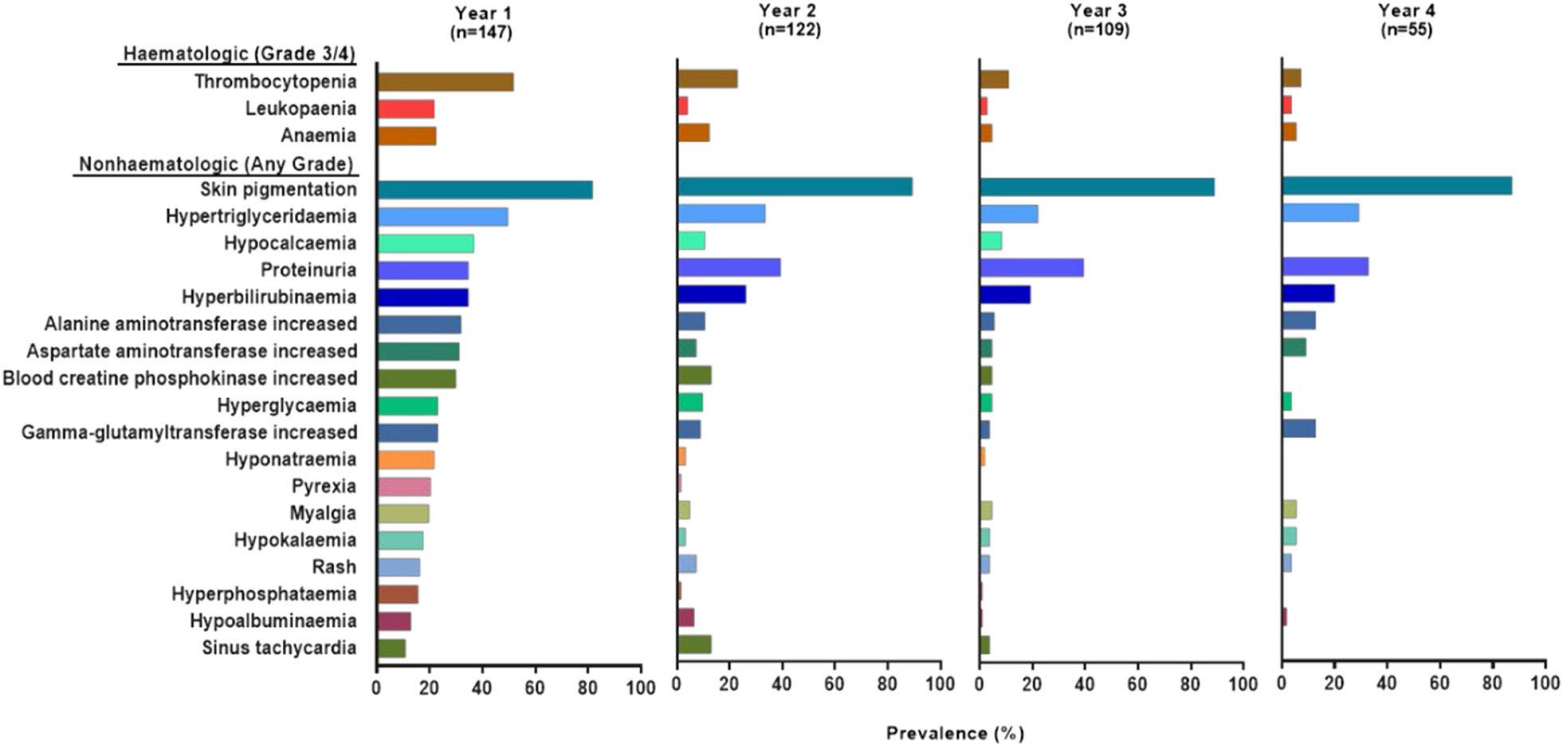
Prevalence of treatment-related adverse events over time

The median age of patients with CVEs was 43 (range, 20–74) years, including two patients with a history of hypertension and three with prior diabetes. All patients with CVEs were required to have temporary olverembatinib treatment suspension and received disease-specific treatment. Most recovered or improved and received olverembatinib treatment at a reduced dose except for one patient who discontinued because of acute myocardial infarction (MI) and one patient who died of pericardial effusion. None of these patients had a Fridericia-corrected QT interval exceeding 500 ms on treatment. Grade 3/4 hematologic TRAEs included thrombocytopenia (51.5%), neutropenia (11.5%), and anemia (23.0%) (Table 3). Myelosuppression tended to occur early, with a median (range) onset time of 28 (4–676) days and a median (range) duration of 36 (7–718) days. Most resolved after temporary treatment suspension or supportive care, including platelet or erythrocyte transfusion or dose adjustment. A total of 11.5% patients received platelet transfusions and 9.7% patients received red blood cell transfusions. Except for skin hyperpigmentation and proteinuria, incidences of TRAEs decreased over time during the follow-up period (Fig. 1). Patients with persistent proteinuria did not observe decreased renal function according to estimated glomerular filtration rate. Serious AEs (SAEs; in ≥ 1% of patients) included thrombocytopenia (9.0%), anemia (6.0%), pneumonia (3.0%), pyrexia or atrial fibrillation (2.0% each); as well as acute MI, cholelithiasis, pericardial effusion, upper-respiratory-tract infection, and urinary-tract infection (1% each). Most SAEs resolved after temporary treatment suspension or dose reduction, which was required in 75 (46.0%) patients at a median of 5 (range 1–36) months.

### Efficacy

#### CML-CP

**Fig. 2:**
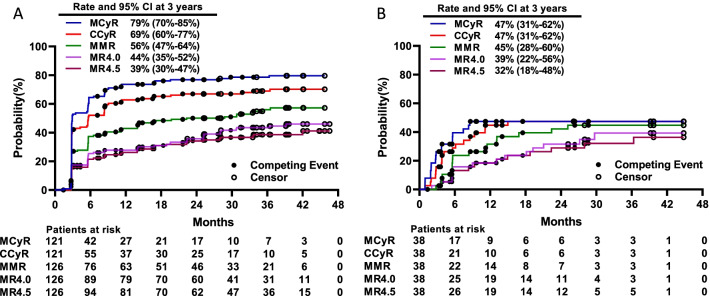
Cumulative incidence of responses in the chronic phase (**A**) and accelerated phase (**B**) MCyR, major cytogenetic response; CCyR, complete cytogenetic response; MMR, major molecular response; MR^4.0^, molecular response 4; MR^4.5^, molecular response 4.5.

**Fig. 3:**
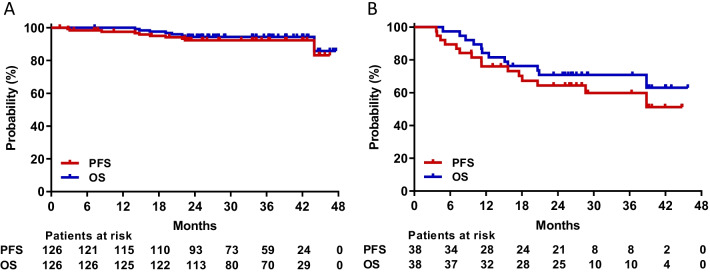
Progression-free survival (PFS) and overall survival (OS) in the chronic phase (**A**) or accelerated phase (**B**)

The median follow-up period of 126 evaluable patients with CML-CP since the start of an effective dose (≥ 30 mg QOD) was 37 (range, 7–58) months. All 84 patients without baseline CHR achieved this endpoint. Of 121 patients without MCyR at baseline, 96 (79.3%) and 84 (69.4%) achieved MCyR and CCyR at a median of 3 (range, 3–36) and 3 (range, 3–37) months, respectively. Among 126 patients, 70 (55.6%) achieved MMR, 56 (44.4%) MR^4.0^, and 49 (38.9%) MR^4.5^ on olverembatinib therapy. Cytogenetic and molecular response rates increased over time (Fig. 2A). The cumulative 3-year incidences of MCyR, CCyR, MMR, MR^4.0^, and MR^4.5^ were 78.6% (95% CI: 70.0%, 85.0%), 69.0% (59·7%, 76·5%), 55.9% (46.5%, 64.4%), 43.5% (34.6%, 52.1%), and 38.6% (30.0%, 47.1%), respectively. The probabilities of sustained MCyR, CCyR, and MMR at 3 years were 77.3% (66.8%, 84.8%), 72.2% (60.4%, 81.1%), and 76.0% (62.1%, 85.3%), respectively. A total of 5 patients progressed to CML-AP (n = 4) or CML-BP (*n* = 1). Seven patients died of disease progression (*n* = 3) and one each of either pericardial effusion, gastric cancer, hepatitis E virus infection (with prior autoimmune hepatitis), or an unknown reason. Probabilities of PFS and OS at 3 years were 92.0% (86.0%, 96.0%) and 94.0% (89.0%, 97.0%), respectively (Fig. 3A).

#### CML-AP

The median follow-up was 27 (range, 5–56) months since the onset of an effective dose. Among 37 patients without baseline MaHR, 29 (78.4%) met this endpoint at a median of 3 (range, 1–7) months; and 27 (73%) experienced CHR at a median of 3 (range, 1–14) months. Of the 38 patients without baseline MCyR, 18 (47.4%) achieved MCyR and CCyR at a median of 3 (range, 1–9) months and 4 (range, 1–15) months. MMR and MR^4.0^ were achieved by 17 (44.7%) and 14 (36.8%) patients, respectively, and MR^4.5^ by 13 (34.2%) patients. Cytogenetic and molecular response rates increased over time (Fig. 2B). The 3-year cumulative incidences of achieving MCyR, CCyR, MMR, MR^4.0^, and MR^4.5^ were 47.4% (30.7%, 62.4%), 47.4% (30.6%, 62.4%), 44.7% (28.2%, 60.0%), 39.3% (22.3%, 56.0%), and 32.1% (17.6%, 47.6%), respectively. The probabilities of sustained MCyR and CCyR at 3 years were 86.0% (55.0%, 97.0%) and 71% (44.0%, 87.0%). A total of 11 patients had CML that progressed to blast phase, and 4 died. Probabilities of PFS and OS at 3 years were 60.0% (41.0%, 74.0%) and 71% (54.0%, 83.0%), respectively (Fig. 3B).

### Responses according to *BCR-ABL1* mutation status via Sanger sequencing

**Fig. 4:**
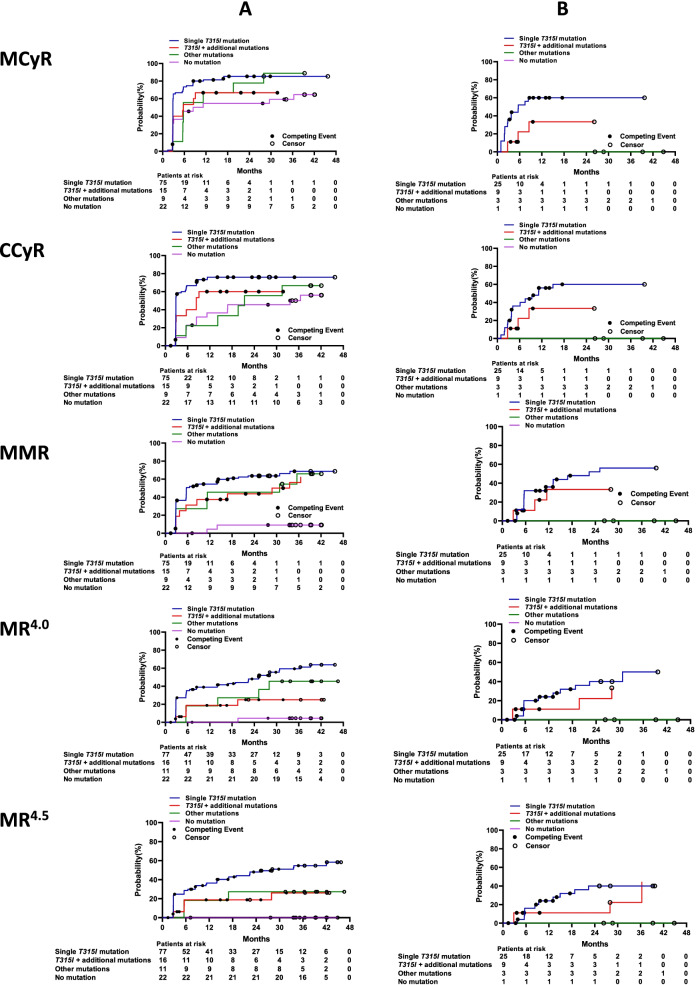
Responses by baseline *BCR-ABL1* mutation status in the chronic phase (**A**) or accelerated phase (**B**) using Sanger sequencing. CCyR, complete cytogenetic response; MCyR, major cytogenetic response; MMR, major molecular response; MR^4.0^, molecular response 4; MR^4.5^, molecular response 4.5

Among the four subgroups with CML-CP evaluated by Sanger sequencing, patients with a single T315I mutation had the highest 3-year cumulative incidences of achieving MCyR (85.3%), CCyR (76.0%), MMR (68.7%), MR^4.0^ (59.3%), and MR^4.5^ (54.7%); those with no *BCR-ABL1* mutation had the lowest cumulative incidences of MCyR (59.1%), CCyR (50.0%), MMR (9.1%), MR^4.0^ (4.5%), and MR^4.5^ (0) (all *P* values among the four subgroups < 0.0001; Fig. 4A). Among patients with CML-AP, those with a single T315I mutation also had the highest 2-year cumulative incidences of MCyR or CCyR (60.0% each), MMR (52.0%), and MR^4.0^ or MR^4.5^ (40.0% each); followed by those with T315I and an additional mutation, who had MCyR, CCyR, or MMR (33.3% each), MR^4.0^ (22.2%), and MR^4.5^ (11.1%). No cytogenetic or molecular response was observed in patients with no *BCR-ABL1* mutation or with other mutations at enrollment (Fig. 4B). We interrogated baseline covariates to evaluate associations with cytogenetic and molecular responses (Table 4).

**Table 4:** Multivariate analysis results of variables associated with treatment responses

	MCyR		CCyR		MMR		MR^4.0^		MR^4.5^	
	HR (95% CI)	*P* value	HR (95% CI)	*P* value	HR (95% CI)	*P* value	HR (95% CI)	*P* value	HR (95% CI)	*P* value
*In 164 evaluable patients*										
Baseline *BCR-ABL1* mutation status by Sanger sequencing		0.10		0.02		0.006		0.006		< 0.001
*Single T315I mutation (ref.)*										
*T315I* + additional mutations	0.6 (0.3–1.0)	0.05	0.6 (0.3–1.1)	0.10	0.8 (0.4–1.3)	0.32	0.4 (0.2–1.0)	0.05	0.5 (0.2–1.1)	0.10
Other mutations	0.7 (0.5–1.2)	0.21	0.6 (0.3–1.0)	0.06	1.0 (0.5–1.8)	0.80	0.6 (0.3–1.3)	0.23	0.4 (0.1–1.6)	0.22
No mutation	0.6 (0.4–1.1)	0.13	0.5 (0.3–0.8)	0.009	1.0 (0.0–0.4)	< 0.001	0.1 (0.0–0.4)	0.005	0.0 (0.0–0.0)	< 0.001
Accelerated phase (ref. chronic phase)	0.5 (0.3–1.0)	0.04	0.5 (0.3–0.9)	0.03	0.6 (0.4–1.0)	0.06	0.7 (0.4–1.3)	0.29	0.8 (0.4–1.5)	0.51
Additional chromosomal abnormalities (ref. none)	0.7 (0.4–1.2)	0.18	0.7 (0.4–1.3)	0.23	0.7 (0.4–1.3)	0.24	0.8 (0.4–1.6)	0.49	0.6 (0.3–1.6)	0.33
Time from diagnosis to olverembatinib treatment, years (continuous)	0.9 (0. 9–1.0)	0.002	1.0 (0.9–1.0)	0.003	1.0 (0.9–1.0)	< 0.001	1.0 (0.9–1.0)	0.002	0.9 (0.8–1.0)	< 0.001
Number of prior TKIs (continuous)	0.8 (0.6–1.1)	0.16	0.7 (0.5–1.1)	0.10	0.7 (0.5–1.0)	0.03	0.7 (0.5–1.1)	0.12	0.8 (0.5–1.2)	0.27
Age (10 years)	0.9 (0.8–1.1)	0.36	0.8 (0.7–1.0)	0.07	0.9 (0.8–1.1)	0.30	0.9 (0.8–1.1)	0.44	1.0 (0.8–1.2)	0.90
*In 118 evaluable patients*										
Baseline *BCR-ABL1* mutation status by next-generation sequencing		0.31		0.13		0.002		< 0.001		< 0.001
*Single T315I mutation (ref.)*										
*T315I* + additional mutations	0.6 (0.3–1.2)	0.15	0.7 (0.3–1.4)	0.26	0.4 (0.1–0.9)	0.03	0.5 (0.2–1.2)	0.13	0.7 (0.3–1.5)	0.35
Other mutations	0.7 (0.4–1.5)	0.40	0.6 (0.3–1.2)	0.14	0.8 (0.4–1.9)	0.67	0.7 (0.3–1.6)	0.42	0.5 (0.1–1.9)	0.29
Compound mutations	0.6 (0.3–1.2)	0.13	0.7 (0.3–1.4)	0.26	0.6 (0.3–1.2)	0.16	0.3 (0.1–1.1)	0.07	0.4 (0.1–1.2)	0.11
No mutation	0.7 (0.3–1.2)	0.15	0.5 (0.3–0.9)	0.02	0.0 (0.0–0.2)	< 0.001	0.0 (0.0–0.0)	< 0.001	0.0 (0.0–0.0)	< 0.001
Accelerated phase (ref. chronic phase)	0.8 (0.7–1.0)	0.10	0.8 (0.6–1.0)	0.03	0.9 (0.7–1.1)	0.21	0.9 (0.7–1.1)	0.45	1.0 (0.8–1.3)	0.82
Additional chromosomal abnormalities (ref. none)	0.3 (0.2–0.7)	0.004	0.4 (0.2–0.8)	0.007	0.4 (0.2–0.8)	0.009	0.7 (0.3–1.4)	0.32	0.8 (0.4–1.7)	0.59
Time from diagnosis to olverembatinib treatment, years (continuous)	0.9 (0.8–1.0)	0.003	1.0 (0.8–1.0)	0.004	0.9 (0.8–1.0)	< 0.001	0.9 (0.8–1.0)	0.003	0.9 (0.8–1.0)	0.002
Number of prior TKIs (continuous)	0.7 (0.4–1.0)	0.03	0.6 (0.4–1.0)	0.02	0.6 (0.4–0.9)	0.01	0.7 (0.4–1.0)	0.08	0.8 (0.5–1.3)	0.33
Age (10 years)	0.8 (0.7–1.0)	0.10	0.8 (0.6–1.0)	0.03	0.9 (0.7–1.1)	0.21	0.9 (0.7–1.1)	0.45	1.0 (0.8–1.3)	0.82

*MCyR* major cytogenetic response, *CCyR* complete cytogenetic response; *CI* confidential interval, *HR* hazard ratio, MMR, major molecular response

*MR*^*4.0*^ molecular response 4, *MR*^*4.5*^ molecular response 4.5, *ref.* reference, *TKI* tyrosine kinase inhibitor

In multivariate analyses, *BCR-ABL1* mutation status before study entry was independently associated with higher cumulative incidences of achieving CCyR (*P* = 0.02), MMR (*P* = 0.006), MR^4.0^ (*P* = 0.006), and MR^4.5^ (*P* < 0.0001). Compared to no mutation or other mutation status, a single T315I mutation showed higher responses (or a trend in this direction). In addition, a longer interval from CML diagnosis to olverembatinib treatment onset, CML-AP rather than CML-CP, and more prior TKIs were significantly associated with lower rates of cytogenetic and molecular responses.

### Responses according to *BCR-ABL1* mutation status via *NGS*

**Fig. 5:**
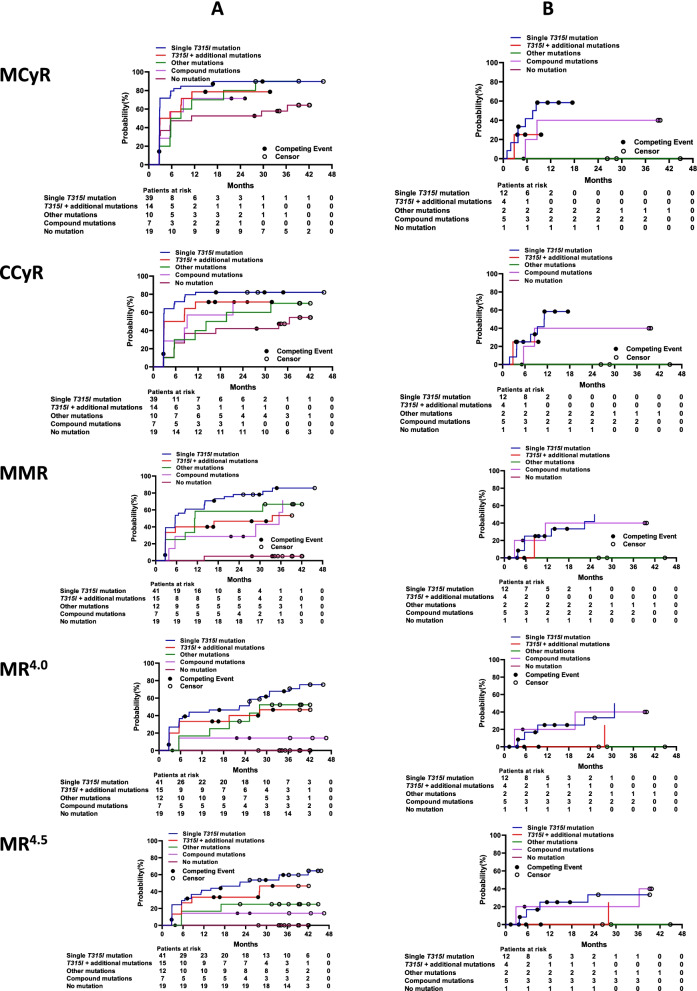
Responses by baseline *BCR-ABL1* mutation status in the chronic phase (**A**) or accelerated phase (**B**) using next-generation sequencing. CCyR, complete cytogenetic response; MCyR, major cytogenetic response; MMR, major molecular response; MR^4.0^, molecular response 4; MR^4.5^, molecular response 4.5

Findings on treatment responses stratified by *BCR-ABL1* mutation status with NGS paralleled those with Sanger sequencing (Fig. 5). Among 12 patients (7 with CML-CP and 5 with CML-AP) with compound mutations (Table 5), 8 (67.0%) had T315I-inclusive compound mutations; and 9 (75.0%), 2 (17.0%), and 1 (8.0%) had 1, 2, and 3 compound mutations, respectively. A total of 7 (58.0%) achieved MMR and 3 (25.0%) MR.^4.5^ By the last follow-up date, 3 patients had progressed to CML-AP or CML-BP and died, and 7 remained on treatment with olverembatinib, of whom one had CCyR and two each had CHR, MMR, or MR.^4.5^

**Table 5:** Characteristics of 12 patients harboring compound mutations at baseline

Pt ID	Disease phase	Prior TKI	Dose, mg	Baseline SS	Baseline NGS (frequency)	Best response	Time to response (mo.)	Response duration (mo.)	Outcome
0401	CP	I D N	40	V299L; F317; F359V	V299L/F359V (60%); V299L/F317L/F359V (3.8%); F317L (33.4%); F359V (3.6%)	MMR	6*	41	Loss of MMR at 36 mo., ongoing in CCyR
0601	CP	I N	40	G250E; T315I; F317L	G250E/F317L (79.1%); G250E (7.9%); T315I (17.5%);	MR^4.5^	3*	47	Ongoing in MR^4.5^
0705	CP	I N	50	T315I; F359V	T315I/F359V (7.1%); T315I (73.0%); F359V (10.4%)	MMR	9**	35	Ongoing in MMR
0722	CP	I	30	K247R; G250E; Y253F; T315I; F359I	K247R/Y253F (55.4%); K247R/T315I (5.5%); K247R/F359I (3.7%); G250E (9.64%); T315I (11.6%); F359I (7.45%)	CHR			Progression to AP and died at 21 mo
0728	CP	I	30	T315I; E459K	T315I/E459K (7.9%); T315I (56.3%); E459K (27.7%)	MMR	3**	1	Consent withdrawal because of breast cancer at 4 mo
1028	CP	I N D	30	L248V; T315I; F359C; E459K	T315I/E459K (6.9%); L248V (6.51%); T315I (13.6%); F359C (5.26%); E459K (59.1%);	MMR	6*	31	Ongoing in MMR
1110	CP	I N	50	T315I; M351T; F359V	M351T/F359V (35.2%); Y253H (5.41%); T315I (4.57%); M351T (8.6%); F359V (22.6%);	CHR			Consent withdrawal at 27 mo
0701	AP	I	30	T315I; F359C	T315I/F359C (4.7%); T315I (24.1%); F359V (51.4%)	MR^4.5^	9**	39	Ongoing in MR^4.5^
0706	AP	I D N	30	E255K; E279A; T315I; F317L	E279A/T315I (4.3%); E279A/F317L (3.4%); E255K (47.1%); F359C (3.09%)	CHR			Progression to AP at 6 mo., to BP at 16 mo., and died at 18 mo
0718	AP	I D	30	T315I; F359I	T315I/F359I (4.4%); T315I (38.1%); F359I (51.3%);	CHR			Ongoing in CHR
1101	AP	I	50	E255K; T315I	E255K/T315I (2.0%); T315I (95.1%);	MR^4.5^	3**	10	Loss of MR^4.5^ at 6 mo., progression to BP and died at 13 mo
1106	AP	I N	50	M244V; H396R	M244V/H396R (60.3%); M244V (39.4%)	CHR			Ongoing in CHR

*AP* accelerated phase, *BP* blast phase, *CP* chronic phase, *CHR* complete hematologic response, *CCyR* complete cytogenetic response, *D* dasatinib

*I* imatinib, *MMR* major molecular response, *MR* molecular response, *NGS* next-generation sequencing, *N* nilotinib, *SS* Sanger sequencing

^*^Achieving PCyR, **Achieving CCyR·

In multivariate analyses, *BCR-ABL1* mutation status before study entry was independently associated with cumulative incidences of achieving MMR (*P* = 0.002), MR^4.0^ (*P* < 0.0001), and MR^4.5^ (*P* < 0.0001; Table 4). Univariate analysis results of variables associated with treatment response are in Additional file 1: Table S4. A single T315I mutation was also significantly associated with higher molecular response rates; compound mutation was comparable to other mutation status except no mutations; and no mutation was associated with lower response rates. In addition, longer intervals from CML diagnosis to olverembatinib treatment onset, CML-AP (rather than CML-CP), more prior TKIs, and increasing age were significantly associated with lower rates of cytogenetic and/or molecular responses.

### Pharmacokinetics and pharmacodynamics

**Fig. 6:**
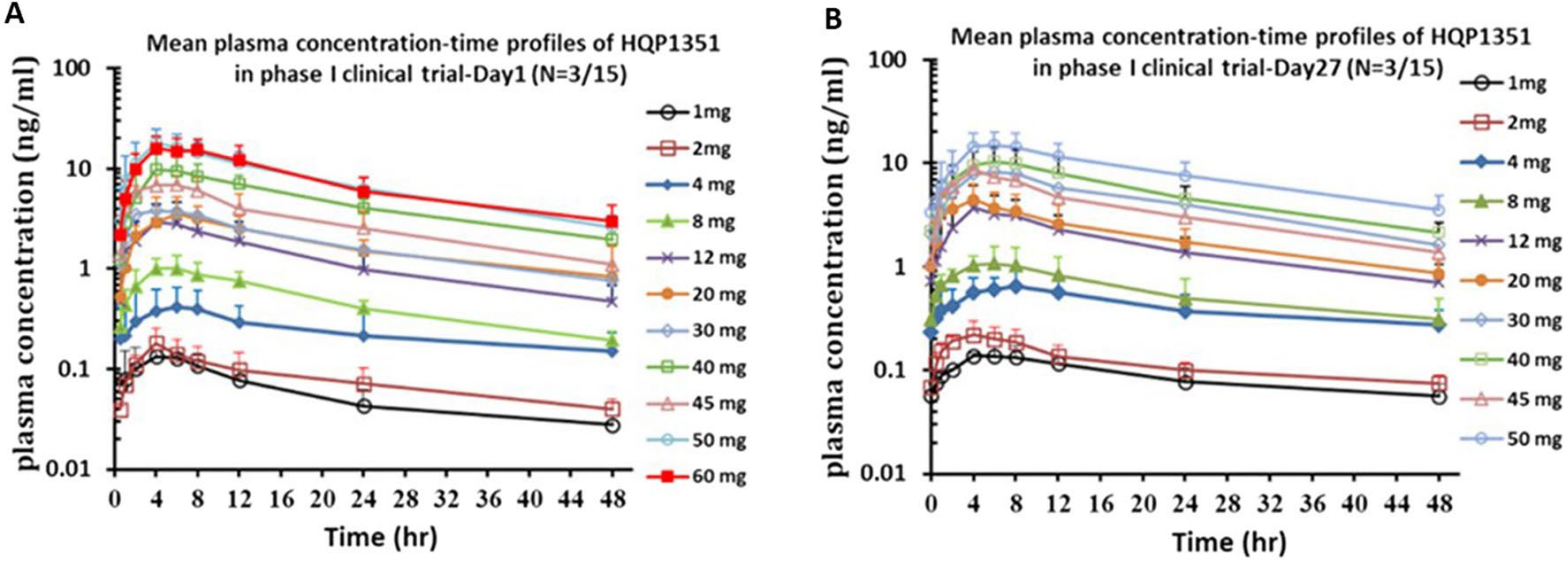
Pharmacokinetics, mean plasma concentration–time curves on treatment Days 1 (**A**) and 27 (**B**)

**Fig. 7:**
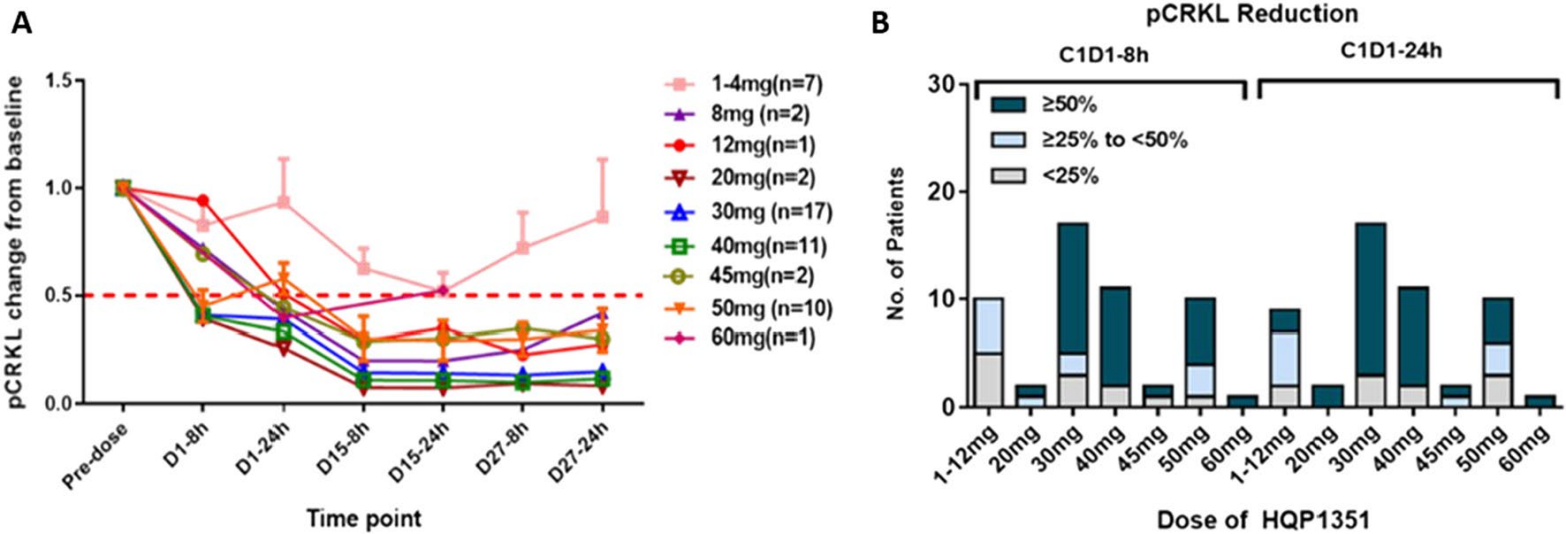
Pharmacodynamics by dose cohorts on Cycle 1 (**A**) and on Day 1 of Cycle 1 (**B**)

Based on plasma concentration–time curves on treatment Days 1 and 27, olverembatinib pharmacokinetics were linear, with mean terminal elimination half-life values (17.5–36.5 h) that are well suited to QOD administration (Fig. 6). Systemic exposure and maximum concentration were approximately dose proportional across olverembatinib doses (1–60 mg QOD). With multiple dosing, slight to moderate accumulation of olverembatinib was observed on C1D27. Significant dose- and time-dependent reductions in pCRKL levels (indicative of BCR-ABL1 inhibition) were observed within 8 h after olverembatinib dosing (30–50 mg QOD) on C1D1 and maintained at steady state on C1D15 and C1D27 (Fig. 7).

## Discussion

We report that novel 3G TKI olverembatinib was well tolerated and efficacious in patients with heavily pretreated, TKI-resistant CML-CP and CML-AP. It exerted robust and durable antitumor activity in patients with a range of potentially clinically challenging *BCR-ABL1* genotypes, including the T315I mutation and compound mutations, which confer treatment resistance or otherwise complicate management with other TKIs [19–22].

The most common and persistent nonhematologic AE with olverembatinib was lentiginous nevus, which presented as scattered benign lesions with no regression that did not require intervention (Additional file 1: Fig. 3). This unique skin change has not been reported in previous TKI cancer studies and might be explained by off-target inhibition of multiple kinases by olverembatinib, although the precise mechanism needs to be elucidated. The most common hematologic TRAE was severe thrombocytopenia, which had a higher incidence than in phase 1 and phase 2 pivotal studies of ponatinib in US studies but similar to that in Japanese patients receiving ponatinib [23, 24]. This TRAE occurred chiefly in the early stage of olverembatinib treatment and may require special attention and intervention, including possible treatment suspension, dose reduction, or platelet transfusion.

Drug-related CVEs are a concern with 3G TKIs. It should be noted that the leading CVE was hypertension (in 13% of patients) and that such events were grade 1 or 2 in 88.5% of patients. During a median follow-up of nearly 3 years, olverembatinib-related CVEs occurred in 32.0% of patients, including a 5.0% incidence of arterial occlusive and venous thrombotic events; this value is lower than the 31.0% reported with ponatinib during a median follow-up of 5 years [25]. However, patients in our study were on average more than 15 years younger and had fewer pre-existing cardiovascular comorbidities (as driven by the eligibility criteria), which would tend to translate into a lower baseline risk of vascular occlusive events. Our trials’ relatively limited follow-up period (vs ponatinib studies) may also contribute to the difference in drug-related CVEs. To further assess the cardiovascular safety profile of olverembatinib, larger patient samples and longer follow-up intervals are required. In future practice, it may be useful to assess and/or stratify cardiovascular risk on TKI treatment via well-validated indices such as the Framingham equations and nomograms.

In general, the effectiveness of olverembatinib was comparable to findings from studies on ponatinib and asciminib [7, 26]. Patients with a single T315I mutation in our study had better cytogenetic and molecular responses to olverembatinib than those with other genotypes. One potential explanation of this finding is that olverembatinib has more specific activity against the T315I mutation, which is consistent with the primary purpose of designing olverembatinib to enhance activity against specific *BCR-ABL1* mutants, including the gatekeeper and genetic variants affecting the P-loop and hinge regions. The effect of the highly specific targeting of the T315I mutation observed with olverembatinib seems to be more pronounced than with ponatinib, which was reported to exert antileukemic activity regardless of baseline *BCR-ABL1* mutation status in the phase 2 ponatinib Ph-positive acute lymphoblastic leukemia (ALL) and CML evaluation (PACE) and Optimizing Ponatinib Treatment in CML (OPTIC) studies [7, 8]; and asciminib, which targets the T315I mutation but requires higher doses in both in vitro and in vivo studies [26, 27].

Drug-resistant compound mutations within the *BCR-ABL1* kinase domain have been reported in many patients receiving sequential TKI therapy. Our study documents definitive responses to olverembatinib in patients with TKI-resistant, heavily pretreated CML-CP or CML-AP with T315I-inclusive or non-T315I-inclusive compound mutations. These responses included MMR and MR^4.5^ in 7 of 12 patients regardless of the type of compound mutation.

Taken together, these findings suggest impressive antileukemic activity of olverembatinib that warrants verification in further studies. In contrast, *BCR-ABL1* compound mutations have been recognized as a potential mechanism of ponatinib treatment failure, which is more commonly detected in CML-AP and Ph^+^ ALL than in CML-CP [7, 19, 28]. On the other hand, non-T315I compound mutants usually retain sensitivity to at least one TKI (especially ponatinib). T315I-inclusive compound mutations confer resistance against all available TKIs, including ponatinib. In general, *BCR-ABL1* compound mutations represent an important clinical challenge.

Potential limitations of our phase 1/2 studies are fourfold. First, we enrolled only Chinese patients with CML and TKI resistance and excluded ponatinib failures. (The 3G TKI is not available in China.) Second, we observed a relatively high number of patients with CML and T315I single mutations and T315I plus additional mutations; conversely, fewer patients had other *BCR-ABL1* mutations or compound mutations. Third, relatively few patients had CML-AP. We adjusted for these potential confounding variables by conducting multivariate analyses. Fourth, the younger age of Chinese patients, which is consistent with overall fewer and less severe comorbidities, might tend to skew both our safety and efficacy results more positively. As such, our conclusions concerning the safety and efficacy of olverembatinib need to be confirmed in future studies with larger sample sizes and more racially and ethnically diverse populations.

## Conclusions

This phase 1/2 study showed that olverembatinib is well tolerated and exhibited robust and durable activity in patients with TKI-resistant CML-CP and CML-AP. In particular, olverembatinib was highly active against *BCR-ABL1*^*T315I*^ mutants and demonstrated encouraging clinical activity against compound mutations. Three Chinese pivotal phase trials (Clinicaltrials.gov NCT03883087, NCT03883100, and NCT04126681) and one US phase 1b trial (NCT04260022) are ongoing.

## Supplementary Information

**Additional file 1. Appendix:** Supplementary tables and figures.

## Data Availability

The datasets generated and/or analyzed during the current study are not publicly available due to this is an ongoing clinical trial containing patients’ information but are available from the sponsor company and corresponding author on reasonable request.

## Abbreviations

AE: Adverse event
ALL: Acute lymphoblastic leukemia
CCyR: Complete cytogenetic response
CHR: Complete hematologic response
CI: Confidential interval
CML: Chronic myeloid leukemia
CML-AP: Accelerated phase chronic myeloid leukemia
CML-CP: Chronic-phase chronic myeloid leukemia
CVE: Cardiovascular event
DLT: Dose-limiting toxicity
ELN: European LeukemiaNet
HR: Hazard ratio
MaHR: Major hematologic response
MCyR: Major cytogenetic response
MI: Myocardial infarction
MMR: Major molecular response
MR^4.0^: 4 Log reduction in BCR-ABL1 transcripts: ≤ 0.01%
MR4.5: (1/10,000) Of cells have the BCR-ABL1 gene
MTD: 4.5 Log reduction in BCR-ABL1 transcripts: ≤ 0.0032% (1/32,000) cells have the BCR-ABL1 gene Maximum tolerated dose
NCI-CTCAE: National Cancer Institute Common Terminology Criteria for Adverse Events
NGS: Next-generation sequencing
OS: Overall survival
PCyR: Partial cytogenetic response
PFS: Progression-free survival
QOD: Every other day
SAEs: Serious adverse events
TKI: Tyrosine kinase inhibitor
TRAEs: Treatment-related adverse events
