# Correction: Olverembatinib (HQP1351), a well-tolerated and effective tyrosine kinase inhibitor for patients with T315I-mutated chronic myeloid leukemia: results of an open-label, multicenter phase 1/2 trial

**DOI:** 10.1186/s13045-023-01414-8

**Published:** 2023-02-20

**Authors:** Qian Jiang, Zongru Li, Yazhen Qin, Weiming Li, Na Xu, Bingcheng Liu, Yanli Zhang, Li Meng, Huanling Zhu, Xin Du, Suning Chen, Yang Liang, Yu Hu, Xiaoli Liu, Yongping Song, Lichuang Men, Zi Chen, Qian Niu, Hengbang Wang, Ming Lu, Dajun Yang, Yifan Zhai, Xiaojun Huang

**Affiliations:** 1 National Clinical Research Center for Hematologic Disease, Peking University People’s Hospital, Peking University Institute of Hematology, No. 11 South Street of Xizhimen, Xicheng District, Beijing, 100044 China; 2 Union Hospital, Tongji Medical College, Huazhong University of Science and Technology, Wuhan, 430022 Hubei China; 3 Department of Hematology, Nanfang Hospital, Southern Medical University, 1838 Guangzhou N Ave, Baiyun, Guangzhou, 510515 Guangdong Province China; 4 State Key Laboratory of Experimental Hematology, National Clinical Research Center for Blood Diseases, Haihe Laboratory of Cell Ecosystem, Institute of Hematology and Blood Diseases Hospital, Chinese Academy of Medical Sciences and Peking Union Medical College, Tianjin, 300020 China; 5 Department of Hematology, The Affiliated Cancer Hospital of Zhengzhou University and Henan Cancer Hospital, Zhengzhou, 450008 Henan China; 6 Department of Hematology, Tongji Hospital of Tongji Medical College, Huazhong University of Science and Technology, Wuhan, 430022 Hubei China; 7 Department of Hematology, West China Hospital of Sichuan University, No. 37 Guoxue Alley, Wuhou District, Chengdu, 610000 Sichuan China; 8 Division of Hematology, Shenzhen Second People’s Hospital, The First Affiliated Hospital of Shenzhen University, 3002 Sungang W Rd, Futian District, Shenzhen, 518000 Guangdong Province China; 9 National Clinical Research Center for Hematologic Diseases, The First Affiliated Hospital of Soochow University, Jiangsu Institute of Hematology, Institute of Blood and Marrow Transplantation, Collaborative Innovation Center of Hematology, Soochow University, Suzhou, China; 10 State Key Laboratory of Oncology in South China, Department of Hematologic Oncology, Collaborative Innovation Center for Cancer Medicine, Sun Yat-Sen University Cancer Center, 651 Dongfeng Road East, Guangzhou, 510060 Guangdong Province China; 11 Guangzhou Healthquest Pharma Co. Ltd., Room F314, GIBI, No. 3 Lanyue Road, Guangzhou, 510663 China; 12 Ascentage Pharma Group Inc, 800 King Farm Blvd Suite 300, Rockville, MD 20850 USA; 13 Ascentage Pharma (Suzhou) Co., Ltd, 218 Xinghu St, Bldg B7, 7Th Floor, Suzhou Industrial Park, Suzhou, 215000 Jiangsu China; 14 State Key Laboratory of Oncology in South China, Collaborative Innovation Center for Cancer Medicine, 651 Dongfeng Road East, Guangzhou, 510060 Guangdong Province China; 15 Beijing Key Laboratory of Hematopoietic Stem Cell Transplantation, No. 11 South Street of Xizhimen, Xicheng District, Beijing, 100044 China; 16 Peking-Tsinghua Center for Life Sciences, No. 11 South Street of Xizhimen, Xicheng District, Beijing, 100044 China; 17 Academy for Advanced Interdisciplinary Studies, Peking University, No. 11 South Street of Xizhimen, Xicheng District, Beijing, 100044 China

**Correction to: Journal of Hematology & Oncology (2022) 15:159** 10.1186/s13045-022-01369-2

The original article [1] presented an incorrect biochemical structure of olverembatinib in Supplementary Figure 1; in addition, the correct structure had indeed already been published.

The figure has since been removed.
