# Supplementary material for: Olverembatinib (HQP1351), a well-tolerated and effective tyrosine kinase inhibitor for patients with T315I-mutated chronic myeloid leukemia: results of an open-label, multicenter phase 1/2 trial
Source: J Hematol Oncol. 2022 Aug 18;15:113. doi: 10.1186/s13045-022-01334-z (PMC9389804; doi:10.1186/s13045-022-01334-z)
Supplement: Supplementary file 1 — Additional file 1. Appendix: Supplementary tables and figures. [file 13045_2022_1334_MOESM1_ESM.docx]

**Appendix: supplementary tables and figures**

**Supplementary Table 1.** Eligibility criteria

**Inclusion:**

- Male or nonpregnant, nonlactating female patients who are 18 years of age or older.
- CML-CP or CML-AP patients with positive Philadelphia chromosome or BCR-ABL1 fusion genes
- Able to understand and voluntarily sign written informed consent form before any study-specific procedure
- ECOG performance status score of ≤ 2
- Life expectancy of ≥ 3 months
- Adequate organ function as indicated by the following laboratory values:
  - Hemoglobin ≥ 8.0 g/dL
  - Leukocyte count ≥ 3.0 × 10^9^/L
  - Platelet count ≥ 75 × 10^9^/L
  - Serum creatinine ≤ 1.5 × upper limit of normal (ULN); or 24 h creatinine clearance ≥ 50 mL/min (Cockcroft-Gault formula) when serum creatinine
    was > 1.5 × ULN
  - Serum albumin ≥ 3.0 g/dL
  - Serum total bilirubin ≤ 1.5 × ULN
  - Aspartate aminotransferase and alanine aminotransferase ≤ 2.5 × ULN
  - Amylase ≤ 1.5 × ULN
  - Lipase ≤ 1.5 × ULN
- Cardiac function index: ejection fraction > 50%; pulmonary arterial systolic pressure
  ≤ 50 mmHg
- Corrected QT interval on electrocardiographic (ECG) evaluation: ≤ 450 ms in men or
  ≤ 470 ms in women
- Men and women of childbearing potential (postmenopausal women must have been amenorrheic for ≥ 12 months to be considered of nonchildbearing potential) and their partners take contraceptive measures considered to be effective from signing the informed consent form to ≥ 120 days after the last dose of olverembatinib
- Willingness and ability to complete study procedures and follow-up examination.

**Exclusion:**

- History of cytotoxic chemotherapy or radiotherapy within 28 days before the first dose; interferon or cytarabine within 14 days
- Failure to recover from treatment adverse events (except for grade > 1 alopecia)
- History of participation in clinical trials of any other investigational drugs within 14 days before the first dose
- Concurrent diseases requiring treatment(s) with potential to interact with olverembatinib
- Some patients with previous treatment with third-generation TKI ponatinib or olverembatinib (or drugs with similar components)
- Malabsorption syndrome or other diseases affecting oral drug absorption
- History of cardiovascular disease, such as hypertension (systolic blood pressure (SBP) > 140 mmHg and/or diastolic blood pressure (DBP) > 90 mmHg) or needing treatments that can prolong the QT ECG interval
- Pulmonary arterial hypertension (according to cardiac ultrasonography, echocardiography, or related clinical symptoms)
- History of serious or severe cardiovascular diseases during previous TKI treatment of CML
- History of autologous or allogeneic stem cell transplantation
- Abnormal coagulation function or history of hemorrhage within 3 months before the first dose of olverembatinib
- History of major surgery (except venous catheterization or bone marrow biopsy) within 14 days before the first dose of olverembatinib
- Required concurrent treatment with immunosuppressive agents, other than corticosteroids (CS) prescribed for a short course of therapy
- Active central nervous system (CNS) disease as evidenced by cytology or pathology. In the absence of CNS disease, lumbar puncture is not required
- Diagnosis of other primary malignancies
- Active symptomatic infections, including HIV and hepatitis
- Known allergy to any components of the investigational drug or its analogues.
- Blood β-human chorionic gonadotropin positive, pregnant, or lactating women, or those expecting pregnancy during the study program (women who are pregnant, nursing, or planning to become pregnant)
- Any condition or illness that would, in the opinion of the investigator, compromise patients' safety or interfere with the evaluation of the efficacy and safety of olverembatinib

**Supplementary Table 2** Adverse events resulting in dose interruption, dose reduction, or treatment discontinuation

|  | Total | Chronic phase | Accelerated phase |
| --- | --- | --- | --- |
| Patient number | 165 | 127 | 38 |
| Adverse events resulting in dose interruption, n (%) | 86 (52.1) | 58 (45.7) | 28 (73.7) |
| Hematologic | 66 (40.0) | 44 (34.6) | 22 (57.9) |
| Thrombocytopenia | 59 (35.8) | 42 (33.1) | 17 (44.7) |
| Leukopenia | 9 (5.5) | 4 (3.1) | 5 (13.2) |
| Anemia | 8 (4.8) | 3 (2.4) | 5 (13.2) |
| Neutropenia | 5 (3.0) | 1 (0.8) | 4 (10.5) |
| Febrile neutropenia | 1 (0.6) | 1 (0.8) | 0 |
| Nonhematologic | 48 (29.1) | 34 (26.8) | 14 (36.8) |
| Increased alanine aminotransferase | 4 (2.4) | 3 (2.4) | 1 (2.6) |
| Increased creatine phosphokinase | 4 (2.4) | 3 (2.4) | 1 (2.6) |
| Hypertriglyceridaemia | 4 (2.4) | 3 (2.4) | 1 (2.6) |
| Increased aspartate aminotransferase | 3 (1.8) | 2 (1.6) | 1 (2.6) |
| Hypocalcemia | 3 (1.8) | 3 (2.4) | 0 |
| Pyrexia | 3 (1.8) | 1 (0.8) | 2 (5.3) |
| Atrial fibrillation | 2 (1.2) | 1 (0.8) | 1 (2.6) |
| Hyperbilirubinaemia | 2 (1.2) | 2 (1.6) | 0 |
| Hypertension | 2 (1.2) | 1 (0.8) | 1 (2.6) |
| Hypokalemia | 2 (1.2) | 0 | 2 (5.3) |
| Increased lipase | 2 (1.2) | 2 (1.6) | 0 |
| Pneumonia | 2 (1.2) | 2 (1.6) | 0 |
| Sinus tachycardia | 2 (1.2) | 1 (0.8) | 1 (2.6) |
| Upper-respiratory-tract infection | 2 (1.2) | 1 (0.8) | 1 (2.6) |
| Acute myocardial infarction | 1 (0.6) | 1 (0.8) | 0 |
| Increased amylase | 1 (0.6) | 1 (0.8) | 0 |
| Anal abscess | 1 (0.6) | 0 | 1 (2.6) |
| Atrial septal defect | 1 (0.6) | 1 (0.8) | 0 |
| Chest pain | 1 (0.6) | 1 (0.8) | 0 |
| Cholecystitis acute | 1 (0.6) | 1 (0.8) | 0 |
| Cholelithiasis | 1 (0.6) | 1 (0.8) | 0 |
| Increased γ-glutamyl transferase | 1 (0.6) | 1 (0.8) | 0 |
| Glaucoma | 1 (0.6) | 1 (0.8) | 0 |
| Hemorrhoids | 1 (0.6) | 1 (0.8) | 0 |
| Hepatocellular injury | 1 (0.6) | 0 | 1 (2.6) |
| Hyperglycaemia | 1 (0.6) | 1 (0.8) | 0 |
| Hyperkalemia | 1 (0.6) | 0 | 1 (2.6) |
| Hyperuricaemia | 1 (0.6) | 1 (0.8) | 0 |
| Hypoesthesia | 1 (0.6) | 1 (0.8) | 0 |
| Hyponatremia | 1 (0.6) | 1 (0.8) | 0 |
| Influenza like illness | 1 (0.6) | 1 (0.8) | 0 |
| Iridocyclitis | 1 (0.6) | 0 | 1 (2.6) |
| Lacunar infarction | 1 (0.6) | 1 (0.8) | 0 |
| Liver disorder | 1 (0.6) | 1 (0.8) | 0 |
| Musculoskeletal pain | 1 (0.6) | 0 | 1 (2.6) |
| Myocardial strain | 1 (0.6) | 1 (0.8) | 0 |
| Pain | 1 (0.6) | 1 (0.8) | 0 |
| Pain in extremity | 1 (0.6) | 1 (0.8) | 0 |
| Pericardial effusion | 1 (0.6) | 0 | 1 (2.6) |
| Peripheral swelling | 1 (0.6) | 1 (0.8) | 0 |
| Proteinuria | 1 (0.6) | 1 (0.8) | 0 |
| Pulmonary arterial hypertension | 1 (0.6) | 1 (0.8) | 0 |
| Increased pulmonary arterial pressure | 1 (0.6) | 1 (0.8) | 0 |
| Pulmonary tuberculosis | 1 (0.6) | 0 | 1 (2.6) |
| Rash | 1 (0.6) | 0 | 1 (2.6) |
| Refraction disorder | 1 (0.6) | 1 (0.8) | 0 |
| Sinus bradycardia | 1 (0.6) | 1 (0.8) | 0 |
| Skin mass | 1 (0.6) | 1 (0.8) | 0 |
| Superior mesenteric artery syndrome | 1 (0.6) | 1 (0.8) | 0 |
| Supraventricular extrasystoles | 1 (0.6) | 0 | 1 (2.6) |
| Swelling | 1 (0.6) | 1 (0.8) | 0 |
| Urinary sediment present | 1 (0.6) | 1 (0.8) | 0 |
| Adverse events resulting in dose reduction, n (%) | 50 (30.3) | 36 (28.3) | 14 (36.8) |
| Hematologic adverse events | 30 (18.2) | 20 (15.7) | 10 (26.3) |
| Thrombocytopenia | 26 (15.8) | 19 (15) | 7 (18.4) |
| Anemia | 3 (1.8) | 2 (1.6) | 1 (2.6) |
| Neutropenia | 3 (1.8) | 2 (1.6) | 1 (2.6) |
| Leukopenia | 2 (1.2) | 0 | 2 (5.3) |
| Nonhaematologic | 21 (12.7) | 16 (12.6) | 5 (13.2) |
| Increased creatine phosphokinase | 2 (1.2) | 2 (1.6) | 0 |
| Hyperbilirubinaemia | 2 (1.2) | 1 (0.8) | 1 (2.6) |
| Hyperglycaemia | 2 (1.2) | 1 (0.8) | 1 (2.6) |
| Hypertension | 2 (1.2) | 2 (1.6) | 0 |
| Retinal-vein occlusion | 2 (1.2) | 2 (1.6) | 0 |
| Acute coronary syndrome | 1 (0.6) | 1 (0.8) | 0 |
| Arteriosclerosis coronary artery | 1 (0.6) | 1 (0.8) | 0 |
| Arthralgia | 1 (0.6) | 0 | 1 (2.6) |
| Asthenia | 1 (0.6) | 1 (0.8) | 0 |
| Atrial fibrillation | 1 (0.6) | 0 | 1 (2.6) |
| Cerebral infarction | 1 (0.6) | 1 (0.8) | 0 |
| Chest discomfort | 1 (0.6) | 1 (0.8) | 0 |
| Dyspnea | 1 (0.6) | 1 (0.8) | 0 |
| Electrocardiogram QT prolonged | 1 (0.6) | 1 (0.8) | 0 |
| Glaucoma | 1 (0.6) | 1 (0.8) | 0 |
| Hypertriglyceridaemia | 1 (0.6) | 1 (0.8) | 0 |
| Hypokalemia | 1 (0.6) | 0 | 1 (2.6) |
| Proteinuria | 1 (0.6) | 1 (0.8) | 0 |
| Seronegative arthritis | 1 (0.6) | 0 | 1 (2.6) |
| Subileus | 1 (0.6) | 1 (0.8) | 0 |
| Adverse events resulting in treatment discontinuation, n (%) | 13 (7.9) | 9 (7.1) | 4 (10.5) |
| Hematologic | 7 (4.2) | 4 (3.1) | 3 (7.9) |
| Thrombocytopenia | 6 (3.6) | 3 (2.4) | 3 (7.9) |
| Febrile neutropenia | 1 (0.6) | 1 (0.8) | 0 |
| Nonhaematologic | 6 (3.6) | 5 (3.9) | 1 (2.6) |
| Acute myocardial infarction | 1 (0.6) | 1 (0.8) | 0 |
| Breast cancer | 1 (0.6) | 1 (0.8) | 0 |
| Lacunar infarction | 1 (0.6) | 1 (0.8) | 0 |
| Pericardial effusion | 1 (0.6) | 0 | 1 (2.6) |
| Proteinuria | 1 (0.6) | 1 (0.8) | 0 |
| Retinal-vein occlusion | 1 (0.6) | 1 (0.8) | 0 |

**Supplementary Table 3** Treatment-related cardiovascular events

| Event | Total | | Chronic phase | | Accelerated phase | |
| --- | --- | --- | --- | --- | --- | --- |
|  | Any grade | G 3/4 | Any grade | G 3/4 | Any grade | G 3/4 |
| Patient number | 165 | 165 | 127 | 127 | 38 | 38 |
| Treatment-related cardiovascular events, n (%) | 53 (32.1) | 19 (11.5) | 44 (34.6) | 15 (11.8) | 9 (23.7) | 4 (10.5) |
| Hypertension | 22 (13.3) | 9 (5.5) | 19 (15.0) | 7 (5.5) | 3 (7.9) | 2 (5.3) |
| Pericardial effusion | 14 (8.5) | 2 (1.2) | 11 (8.7) | 1 (0.8) | 3 (7.9) | 1 (2.6) |
| Ventricular extrasystoles | 7 (4.2) | 0 | 7 (5.5) | 0 | 0 | 0 |
| Atrial fibrillation | 5 (3.0) | 2 (1.2) | 2 (1.6) | 1 (0.8) | 3 (7.9) | 1 (2.6) |
| Supraventricular extrasystoles | 5 (3.0) | 0 | 3 (2.4) | 0 | 2 (5.3) | 0 |
| Retinal-vein occlusion | 3 (1.8) | 2 (1.2) | 3 (2.4) | 2 (1.6) | 0 | 0 |
| Palpitations | 2 (1.2) | 0 | 1 (0.8) | 0 | 1 (2.6) | 0 |
| Acute coronary syndrome | 1 (0.6) | 1 (0.6) | 1 (0.8) | 1 (0.8) | 0 | 0 |
| Acute myocardial infarction | 1 (0.6) | 1 (0.6) | 1 (0.8) | 1 (0.8) | 0 | 0 |
| Angina pectoris | 1 (0.6) | 0 | 1 (0.8) | 0 | 0 | 0 |
| Arrhythmia | 1 (0.6) | 1 (0.6) | 1 (0.8) | 1 (0.8) | 0 | 0 |
| Arteriosclerosis coronary artery | 1 (0.6) | 1 (0.6) | 1 (0.8) | 1 (0.8) | 0 | 0 |
| Atrial tachycardia | 1 (0.6) | 0 | 1 (0.8) | 0 | 0 | 0 |
| Cardiomegaly | 1 (0.6) | 0 | 1 (0.8) | 0 | 0 | 0 |
| Cerebral infarction | 1 (0.6) | 1 (0.6) | 1 (0.8) | 1 (0.8) | 0 | 0 |
| Lacunar infarction | 1 (0.6) | 1 (0.6) | 1 (0.8) | 1 (0.8) | 0 | 0 |

**Supplementary Table 4** Univariate analysis results of variables associated with treatment response

|  | MCyR |  | CCyR |  | MMR |  | MR^4.0^ |  | MR^4.5^ |  |
| --- | --- | --- | --- | --- | --- | --- | --- | --- | --- | --- |
| Variables | HR  (95% CI) | P value | HR  (95% CI) | P value | HR  (95% CI) | P value | HR  (95% CI) | P value | HR  (95% CI) | P value |
| In 164 evaluable patients |  |  |  |  |  |  |  |  |  |  |
| Baseline BCR-ABL1 mutation status by SS |  | 0.010 |  | 0.004 |  | 0.003 |  | 0.002 |  | <0.0001 |
| Single *T315I* mutation (ref.) |  |  |  |  |  |  |  |  |  |  |
| *T315I* + additional mutations | 0.5  (0.3-0.8) | 0.01 | 0.5  (0.3-1.0) | 0.03 | 0.7  (0.4-1.2) | 0.15 | 0.4  (0.2-0.9) | 0.02 | 0.4  (0.2-1.0) | 0.04 |
| Other mutations | 0.5  (0.3-0.9) | 0.03 | 0.4  (0.2-0.9) | 0.02 | 0.6  (0.3-1.4) | 0.25 | 0.5  (0.2-1.2) | 0.12 | 0.3  (0.1-1.1) | 0.07 |
| No mutation | 0.6  (0.3-1.0) | 0.06 | 0.5  (0.3-0.8) | 0.007 | 0.1 (0.0-0.3) | <0.001 | 0.1  (0.0-0.4) | 0.003 | 0.0  (0.0-0.0) | <0.0001 |
| Accelerated phase (ref. chronic phase) | 0.5  (0.3-0.8) | 0.009 | 0.6  (0.3-0.9) | 0.02 | 0.7  (0.4-1.1) | 0.15 | 0.8  (0.4-1.3) | 0.36 | 0.8  (0.5-1.5) | 0.51 |
| Additional chromosomal abnormalities  (ref. none) | 0.5  (0.3-0.8) | 0.009 | 0.5  (0.3-0.9) | 0.02 | 0.6  (0.3-1.1) | 0.08 | 0.6  (0.3-1.2) | 0.17 | 0.6  (0.3-1.2) | 0.13 |
| Time from diagnosis to olverembatinib treatment, years (continuous) | 0.9  (0.9-1.0) | <0.0001 | 0.9  (0.9-0.9) | <0.0001 | 0.9  (0.8-0.9) | <0.0001 | 0.9  (0.8-0.9) | <0.0001 | 0.9  (0.8-0.9) | <0.0001 |
| Number of prior TKIs (continuous) | 0.7  (0.5-0.9) | 0.010 | 0.6  (0.5-0.8) | 0.001 | 0.5 (0.3-0.7) | <0.0001 | 0.5 (0.4-0.8) | <0.001 | 0.5  (0.4-0.8) | 0.001 |
| Age (10 years) | 0.9  (0.8-1.1) | 0.20 | 0.8  (0.7-1.0) | 0.02 | 0.8  (0.7-1.0) | 0.08 | 0.9  (0.7-1.1) | 0.15 | 1.0  (0.8-1.2) | 0.68 |
| In 118 evaluable patients |  |  |  |  |  |  |  |  |  |  |
| Baseline BCR-ABL1 mutation status by NGS |  | 0.09 |  | 0.03 |  | 0.003 |  | <0.0001 |  | <0.0001 |
| Single *T315I* mutation (ref.) |  |  |  |  |  |  |  |  |  |  |
| *T315I* + additional mutations | 0.6  (0.3-1.3) | 0.19 | 0.7  (0.3-1.4) | 0.27 | 0.5  (0.2-1.0) | 0.05 | 0.5  (0.2-1.2) | 0.12 | 0.7  (0.3-1.4) | 0.30 |
| Other mutations | 0.6  (0.3-1.0) | 0.06 | 0. 5  (0.2-0.9) | 0.03 | 0.6  (0.3-1.3) | 0.20 | 0.5  (0.2-1.2) | 0.14 | 0.3  (0.1-1.1) | 0.06 |
| Compound mutations | 0.5 (0.2-1.0) | 0.05 | 0.5  (0.2-1.1) | 0.10 | 0.5  (0.3-1.1) | 0.08 | 0.3  (0.1-1.0) | 0.05 | 0.4  (0.1-1.2) | 0.08 |
| No mutation | 0.5  (0.3-1.0) | 0.05 | 0.4  (0.2-0.8) | 0.008 | 0.0  (0.0-0.3) | <0.001 | 0.0  (0.0-0.0) | <0.0001 | 0.0  (0.0-0.0) | <0.0001 |
| Accelerated phase (ref. chronic phase) | 0.4  (0.2-0.7) | 0.002 | 0.4  (0.2-0.8) | 0.01 | 0.5  (0.3-1.0) | 0.05 | 0.6  (0.3-1.3) | 0.24 | 0.7  (0.3-1.5) | 0.36 |
| Additional chromosomal abnormalities  (ref. none) | 0.5  (0.3-0.9) | 0.02 | 0.5  (0.3-1.0) | 0.04 | 0.6  (0.3-1.1) | 0.11 | 0.6  (0.3-1.3) | 0.22 | 0.5  (0.2-1.3) | 0.17 |
| Time from diagnosis to olverembatinib treatment, years (continuous) | 0.9  (0.8-0.9) | <0.0001 | 0.9  (0.8-0.9) | <0.0001 | 0.8  (0.8-0.9) | <0.0001 | 0.8  (0.8-0.9) | <0.001 | 0.8  (0.8-0.9) | <0.001 |
| Number of prior TKIs (continuous) | 0.6  (0.4-0.8) | 0.003 | 0.5  (0.4-0.7) | <0.001 | 0.5  (0.3-0.6) | <0.0001 | 0.5  (0.3-0.7) | <0.001 | 0.5  (0.3-0.8) | 0.002 |
| Age (10 years) | 0.8  (0.7-1.0) | 0.09 | 0.8  (0.6-1.0) | 0.03 | 0.8  (0.6-1.0) | 0.06 | 0.8  (0.6-1.0) | 0.10 | 0.9  (0.7-1.2) | 0.60 |

**Supplementary Table 5** Characteristics of second-line treatment patients

|  | Total | Chronic phase | Accelerated phase |
| --- | --- | --- | --- |
| Patient number | 30 | 21 | 9 |
| Age (y), median (range) | 43.5 (20-64) | 48 (20-64) | 31 (26-55) |
| Male, n (%) | 21 (70.0) | 12 (57.1) | 9 (100.0) |
| ECOG performance status, n (%) | | | |
| 0 | 16 (53.3) | 10 (47.6) | 6 (66.7) |
| 1 | 14 (46.7) | 11 (52.4) | 3 (33.3) |
| Time from diagnosis to olverembatinib treatment (y), median (range) | 1.62 (0.3-14.6) | 1.64 (0.6-14.6) | 1.61 (0.3-11.6) |
| Prior TKI, n (%) | | | |
| Imatinib | 22 (73.3) | 16 (76.2) | 6 (66.7) |
| Nilotinib | 6 (20.0) | 4 (19.0) | 2 (22.2) |
| Dasatinib | 2 (6.7) | 1 (4.8) | 1 (11.1) |
| BCR-ABL1 mutation status by Sanger sequencing, n (%) | | | |
| No mutation | 1 (3.3) | 1 (4.8) | 0 |
| T315I single mutation | 19 (63.3) | 15 (71.4) | 4 (44.4) |
| T315I + other mutations | 9 (30) | 4 (19) | 5 (55.6) |
| Other mutations | 1 (3.3) | 1 (4.8) | 0 |
| BCR-ABL1 mutation status by next-generation sequencing, n (%) | | | |
| No mutation | 1 (3.3) | 1 (4.8) | 0 |
| T315I single mutation | 10 (33.3) | 9 (42.9) | 1 (11.1) |
| T315I + additional mutations | 6 (20.0) | 4 (19) | 2 (22.2) |
| Other mutations | 2 (6.7) | 2 (9.5) | 0 |
| Compound mutations | 4 (13.3) | 2 (9.5) | 2 (22.2) |
| ACA, n (%) |  |  |  |
| Yes | 6 (20.0) | 2 (9.5) | 4 (44.4) |
| No | 24 (80.0) | 19 (90.5) | 5 (55.6) |
| ACA, additional chromosomal abnormalities; ECOG, Eastern Cooperative Oncology Group; TKI, tyrosine kinase inhibitor. | | | |

**Supplementary Table 6** Summary of T315I+additional mutations at baseline by Sanger sequencing

|  | Total | Chronic phase | Accelerated phase |
| --- | --- | --- | --- |
| Patient number | 165 | 127 | 38 |
| T315I+E255K | 3 (1.8%) | 1 (0.8%) | 2 (5.3%) |
| T315I+E450A | 1 (0.6%) | 0 | 1 (2.6%) |
| T315I+E459K | 2 (1.2%) | 1 (0.8%) | 1 (2.6%) |
| T315I+E459K+F359C+L248V | 1 (0.6%) | 1 (0.8%) | 0 |
| T315I+E459Q | 1 (0.6%) | 1 (0.8%) | 0 |
| T315I+F317L | 3 (1.8%) | 2 (1.6%) | 1 (2.6%) |
| T315I+F317L+E255K+E279A | 1 (0.6%) | 0 | 1 (2.6%) |
| T315I+F317L+G250E | 1 (0.6%) | 1 (0.8%) | 0 |
| T315I+F317L+G250E+M351T | 1 (0.6%) | 1 (0.8%) | 0 |
| T315I+F359C | 1 (0.6%) | 0 | 1 (2.6%) |
| T315I+F359I | 1 (0.6%) | 0 | 1 (2.6%) |
| T315I+F359I+G250E+K247R+Y253F | 1 (0.6%) | 1 (0.8%) | 0 |
| T315I+F359V | 2 (1.2%) | 2 (1.6%) | 0 |
| T315I+F359V+M351T | 1 (0.6%) | 1 (0.8%) | 0 |
| T315I+M244V | 2 (1.2%) | 1 (0.8%) | 1 (2.6%) |
| T315I+M244V+E255V | 1 (0.6%) | 1 (0.8%) | 0 |
| T315I+Y253H | 2 (1.2%) | 2 (1.6%) | 0 |

**Supplementary Table 7** Patient characteristics of phase 1 and phase 2 study

|  | **Phase 1 study** | | | **Phase 2 study** | |
| --- | --- | --- | --- | --- | --- |
|  | Total | Chronic phase | Accelerated phase | Chronic phase | Accelerated phase |
| Patient number | 101 | 86 | 15 | 41 | 23 |
| Age (y), median (range) | 40 (20-64) | 40.5 (20-64) | 37 (25-51) | 47 (22-70) | 41 (21-74) |
| Male, n (%) | 71 (70.3%) | 58 (67.4%) | 13 (86.7%) | 21 (51.2%) | 18 (78.3%) |
| ECOG | | | | | |
| 0 | 75 (74.3%) | 61 (70.9%) | 14 (93.3%) | 21 (51.2%) | 3 (13.0%) |
| 1 | 24 (23.8%) | 23 (26.7%) | 1 (6.7%) | 20 (48.8%) | 20 (87.0%) |
| Not done | 2 (2.0) | 2 (2.3) | 0 | 0 | 0 |
| Time from diagnosis to olverembatinib treatment (y), median (range) | 5.95 (0.3-15.2) | 5.64 (0.6-15.2) | 8.04 (0.3-14.7) | 5.31 (0.6-23.2) | 4.96 (0.4-10.2) |
| Number of lines of prior TKI therapy, n (%) | | | | | |
| 1 | 17 (16.8%) | 12 (14.0%) | 5 (33.3%) | 9 (22.0%) | 4 (17.4%) |
| 2 | 51 (50.5%) | 46 (53.5%) | 5 (33.3%) | 25 (61.0%) | 14 (60.9%) |
| ≥3 | 33 (32.7%) | 28 (32.6%) | 5 (33.3%) | 7 (17.1%) | 5 (21.7%) |
| Prior TKIs, n (%) | | | | | |
| Imatinib | 12 (11.9%) | 8 (9.3%) | 4 (26.7%) | 8 (19.5%) | 2 (8.7%) |
| Imatinib/dasatinib | 33 (32.7%) | 30 (34.9%) | 3 (20.0%) | 17 (41.5%) | 10 (43.5%) |
| Imatinib/nilotinib | 16 (15.8%) | 14 (16.3%) | 2 (13.3%) | 8 (19.5%) | 2 (8.7%) |
| Imatinib/dasatinib/nilotinib | 33 (32.7%) | 28 (32.6%) | 5 (33.3%) | 7 (17.1%) | 5 (21.7%) |
| Nilotinib | 5 (5.0%) | 4 (4.7%) | 1 (6.7%) | 0 | 1 (4.3%) |
| Dasatinib | 0 | 0 | 0 | 1 (2.4%) | 1 (4.3%) |
| Dasatinib/nilotinib | 2 (2.0%) | 2 (2.3%) | 0 | 0 | 2 (8.7%) |
| BCR-ABL1 mutation status by Sanger sequencing, n (%) | | | | | |
| No mutation | 24 (23.8%) | 23 (26.7%) | 1 (6.7%) | 0 | 0 |
| T315I single mutation | 46 (45.5%) | 40 (46.5%) | 6 (40.0%) | 37 (90.2%) | 19 (82.6%) |
| T315I + additional mutations | 17 (16.8%) | 12 (14.0%) | 5 (33.3%) | 4 (9.8%) | 4 (17.4%) |
| Other mutations | 14 (13.9%) | 11 (12.8%) | 3 (20.0%) | 0 | 0 |
| BCR-ABL1 mutation status by next-generation sequencing, n (%)* | | | | | |
| No mutation | 20 (21.3%) | 19 (23.5%) | 1 (7.7%) | 0 | 0 |
| T315I single mutation | 33 (35.1%) | 30 (37.0%) | 3 (23.1%) | 11 (84.6%) | 9 (81.8%) |
| T315I + additional mutations | 16 (17.0%) | 14 (17.3%) | 2 (15.4%) | 1 (7.7%) | 2 (18.2%) |
| Other mutations | 13 (13.8%) | 11 (13.6%) | 2 (15.4%) | 1 (7.7%) | 0 |
| Compound mutations | 12 (12.8%) | 7 (8.6%) | 5 (38.5%) | 0 | 0 |
| ACA, n(%) | | | | | |
| Yes | 17 (16.8%) | 9 (10.5%) | 8 (53.3%) | 1 (2.4%) | 10 (43.5%) |
| No | 84 (83.2%) | 77 (89.5%) | 7 (46.7%) | 40 (97.6%) | 13 (56.5%) |
| ACA, additional chromosomal abnormalities; ECOG, Eastern Cooperative Oncology Group; TKI, tyrosine kinase inhibitor.  * 94 patients in phase 1 study performed next-generation sequencing to identify BCR-ABL1 mutations, 81 patients with chronic phase and 13 patients with accelerated phase; 24 patients in phase 2 study performed next-generation sequencing to identify BCR-ABL1 mutations, 13 patients with chronic phase and 11 patients with accelerated phase. | | | | | |

**
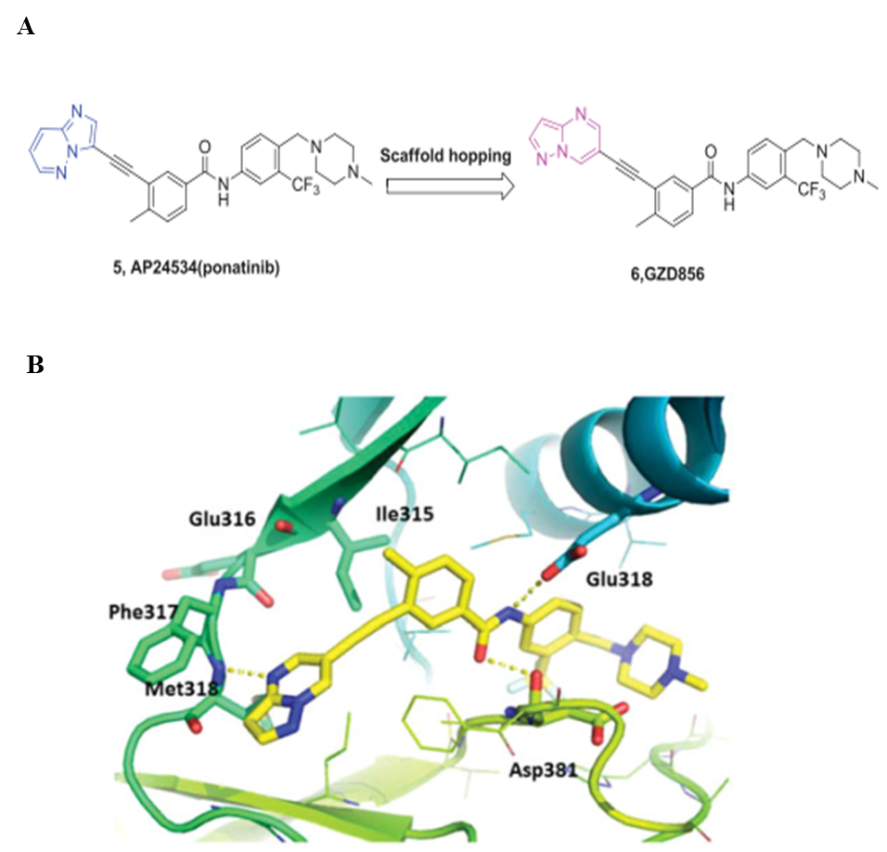
**

**Supplementary Figure 1** Design of olverembatinib and predicted binding mode of olverembatinib and BCR-ABL1 *T3151.*

**A)** Design of olverembatinib (HQP1351; GZD856) as a new BCR-ABL1 inhibitor by scaffold hopping based on ponatinib. **B)** Predicted binding mode of GZD856 with BCR-ABL1*T315I*. Hydrogen bonds are indicated by yellow hatched lines to key amino acids. Reprinted (adapted) with permission from (Ren X et al. Identification of GZD824 as an orally bioavailable inhibitor that targets phosphorylated and nonphosphorylated breakpoint cluster region–Abelson (BCR-ABL1) kinase and overcomes clinically acquired mutation-induced resistance against imatinib. J Med Chem 2013; 56:879-894. Copyright (^©^2013) American Chemical Society.


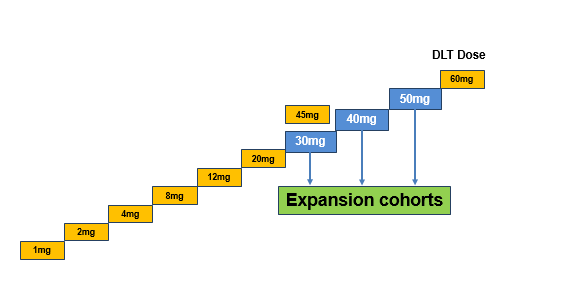


**Supplementary Figure 2** Study design.

Olverembatinib was administered orally on alternate days, according to the schema shown above
(one cycle = 28 days). The first dose was given on October 26, 2016.
Other key points:

**Dose escalation**

- 3+3 dose escalation design across 11 dose cohorts
- A total of 15 subjects in the maximum tolerated dose (MTD) dose cohort
- Intrapatient dose escalation permitted unless the olverembatinib regimen exceeded the MTD

**Dose expansion**

- Up to 60 subjects

**
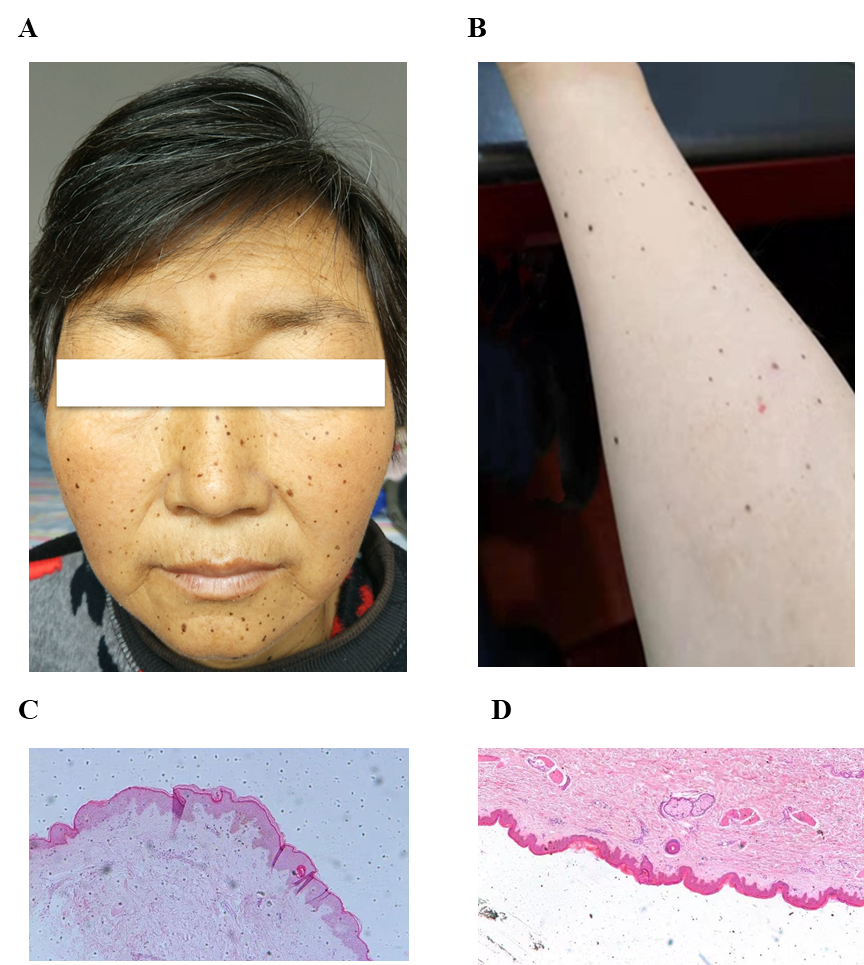
**

**Supplementary Figure 3** Skin pigmentation (A, B) and lentiginous nevus (C,D, H-E stain, ×40 magnification). Pathologic description: microscopically, the rash on the left face was a brownish blotch with different sizes, with more dark brown spots and spherules (some of which were pseudo reticular). The surrounding hair follicles showed a similar appearance but were lighter in hue.
